# (2,9-Dimethyl-1,10-phenanthroline)bis­[2-(pyridin-2-yl)phen­yl]iridium(III) hexa­fluoro­phosphate and (2,9-dimethyl-1,10-phenanthroline)bis­[5-methyl-2-(pyridin-2-yl)phen­yl]iridium(III) hexa­fluoro­phosphate–diethyl ether–aceto­nitrile (1/0.61/0.78)

**DOI:** 10.1107/S2056989024012039

**Published:** 2025-01-14

**Authors:** Trevor J. Gienau, Malachi Clay, William W. Brennessel, Carly R. Reed

**Affiliations:** a Department of Chemistry and Biochemistry State University of New York at Brockport Brockport NY 14420 USA; b Department of Chemistry 120 Trustee Road University of Rochester,Rochester NY 14627 USA; University of Durham, United Kingdom

**Keywords:** crystal structure, iridium, 2-phenyl­pyridine, 2-(4-methyl­phen­yl)pyridine, 2,9-dimethyl-1,10-phenanthroline

## Abstract

The title compounds, [Ir(C_14_H_12_N_2_)(C_11_H_8_N)_2_]PF_6_ (**1**) and [Ir(C_14_H_12_N_2_)(C_12_H_10_N)_2_]PF_6_·0.61C_2_H_10_O·0.78CH_3_CN (**2**), crystallize in the space groups *Pbca* and *P*1, respectively, each structure containing one monocationic Ir complex and one PF_6_^−^ anion in the asymmetric unit. The anion and solvent in compound **2** are disordered. The Ir—N(phenanthroline) bond lengths of *ca*. 2.21 Å indicate a greater steric effect of the 2,9-dimethyl-1,10-phenanthroline ligand compared to 1,10-phenanthroline. Both structures show offset parallel inter­molecular π–π inter­actions between the pyridine rings of the phenanthroline ligands, and that of **1** also exhibits similar inter­actions between the phenyl and pyridine rings of the phenyl­pyridine ligands.

## Abstract 2

The title compounds exhibit Ir—C and Ir—N bond lengths typical of cyclo­metallated iridium compounds with phenyl­pyridine ligands. Methyl­ation of the phenanthroline ligand leads to longer Ir—N bond lengths compared to the unmethyl­ated analog.

## 1. Chemical context

Cyclo­metallated iridium complexes of the form [Ir(C^N)_2_(N^N)](PF_6_), where C^N and N^N are aromatic chelating ligands, have gained inter­est due to their long-lived luminescence and high photostability. These lumiphores have found application in optoelectronics, bioimaging, biosensing, and cancer treatments (Mills *et al.*, 2018 ▸; Xu *et al.*, 2021 ▸; Berrones Reyes *et al.*, 2021 ▸; Ho *et al.*, 2020 ▸; Jing *et al.*, 2024 ▸). Understanding how changes to the coordination environment around the iridium metal center impact the structure of the mol­ecule is crucial, as structural changes influence the properties of these complexes. Modifications to iridium complexes have been shown to affect: emission energy, emission quantum yield, excited state lifetime, solubility, biomolecule selectivity, the strength of the inter­action with a biomolecule, and luminescence enhancement in the presence of a biomolecule (Mills *et al.*, 2018 ▸; Ma *et al.*, 2015 ▸; Lin *et al.*, 2014 ▸; He *et al.*, 2013 ▸; Castor *et al.*, 2015 ▸).

In this study, we examine the structures of compounds **1** and **2**, whose iridium cations were previously investigated for their application in light-emitting electrochemical cells and G-quadruplex luminescent turn-on detection platforms (Moon & Choe, 2013 ▸; Ma *et al.*, 2014 ▸). Compound **1** was previously crystallized in the *P*2_1_/*c* space group as a deuterated chloro­form solvate, **3** (Batsanov, 2017*a* ▸). The cation of **1** was also crystallized with a different counter-ion (Ma *et al.*, 2016 ▸).
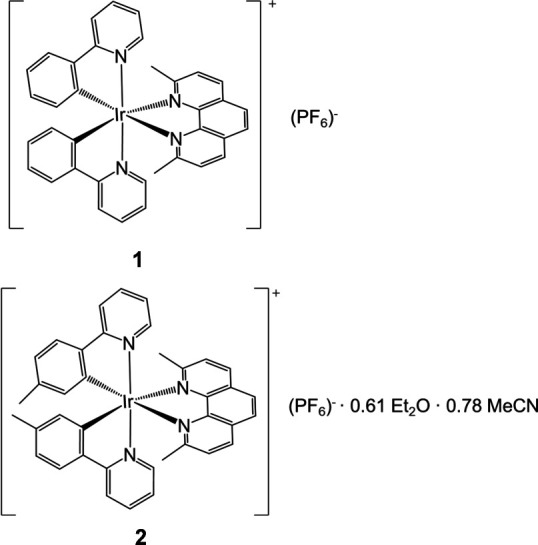


## 2. Structural commentary

**Figure 1:**
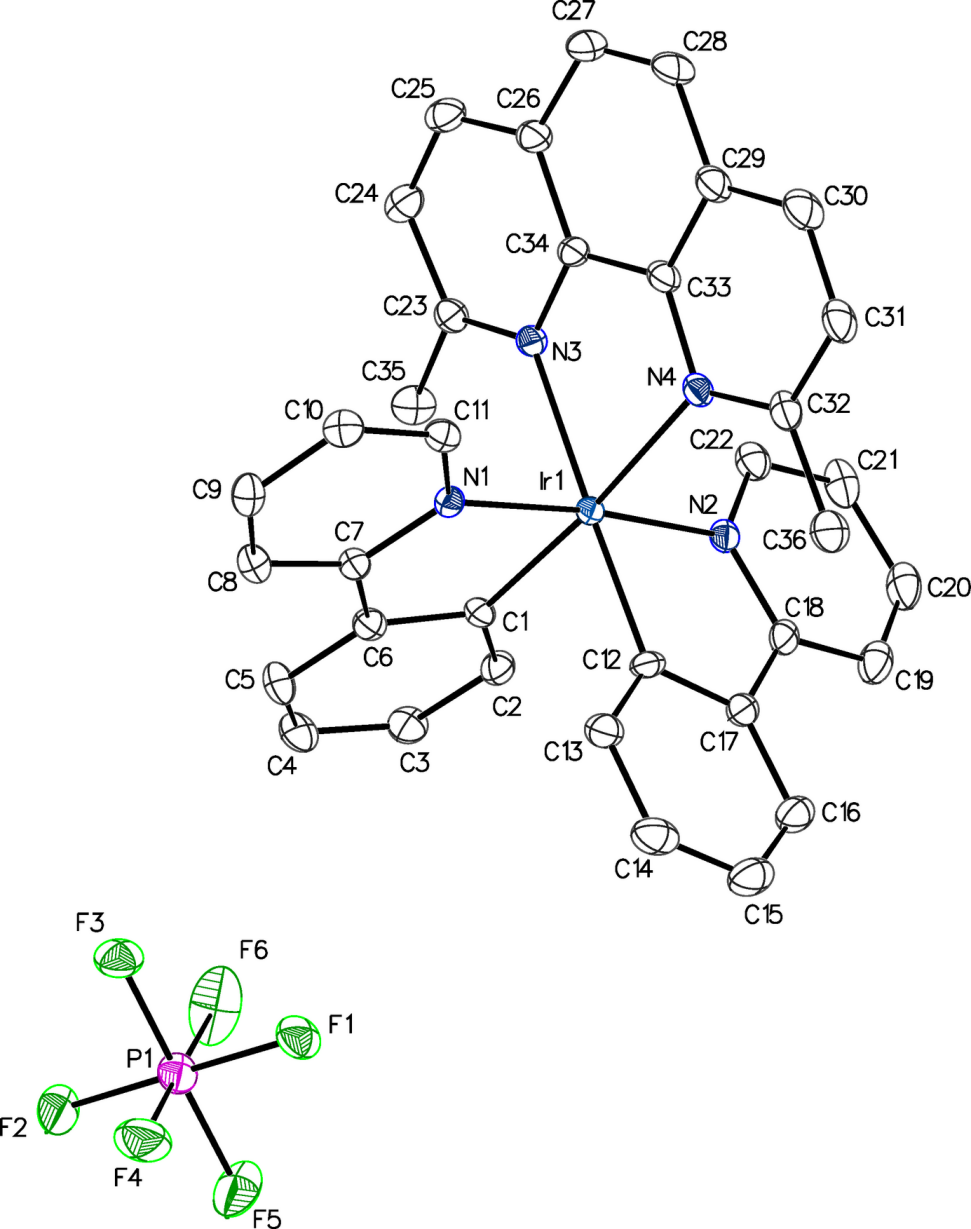
Anisotropic displacement ellipsoid plot of **1** drawn at the 50% probability level, with H atoms omitted. The PF_6_^−^ anion has been shifted with symmetry operation −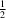 + *x*, *y*, 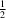 − *z*.

The mol­ecular structures of **1** and **2** are shown in Figs. 1 ▸ and 2 ▸, while Tables 1 ▸ and 2 ▸ list bond lengths and angles involving the Ir atoms, for **1** and **2**, respectively, and Table 3 ▸ compares these with the corresponding ones in **3** and in the fully unmethyl­ated analog, **4** (Batsanov, 2017*b* ▸). The Ir—C (*ca.* 2.02 Å) and Ir—N_(CN)_ (*ca.* 2.05 Å) bond lengths are similar across all four compounds, indicating methyl­ation at the 5-position of the phenyl­pyridine ligand does not have a steric impact. However, the Ir—N_(NN)_ distances increase with methyl­ation at the 2- and 9-positions of the phenanthroline ligand, to *ca*. 2.21 Å in compounds **1**–**3**, compared to *ca*. 2.14 Å in the unmethyl­ated reference compound **4**. This is similar to other previously published compounds methyl­ated in the 2- and 9-positions (Graf *et al.*, 2014 ▸; Graf, Böttcher *et al.*, 2021 ▸). The steric impact of the methyl groups on Ir—N_(NN)_ bond distances is unique to the 2- and 9-positions. Methyl­ation at any other position of the phenanthroline ligand results in Ir—N_(NN)_ bond distances similar to those of **4** (Graf *et al.*, 2020 ▸; Graf, Czerwieniec *et al.*, 2021 ▸). The steric impact of methyl­ation is also reflected in the bond angles displayed in Table 3 ▸: the C—Ir—N_(NN)_ bond angles in **1**–**3** are wider, while the C—Ir—C angles are narrower, than those in **4**.

## 3. Supra­molecular features

In the structures of **1** and **2** there are a number of C—H⋯F—P contacts (Tables 4 ▸ and 5 ▸). Those with H⋯F distances shorter than the sum of the van der Waals radii (2.56 Å; Rowland & Taylor, 1996 ▸) are listed in Tables 4 ▸ and 5 ▸, respectively, for **1** and **2**. These attractions are likely very weak, of the same order of energies as in van der Waals complexes (Howard *et al.*, 1996 ▸).

**Figure 3:**
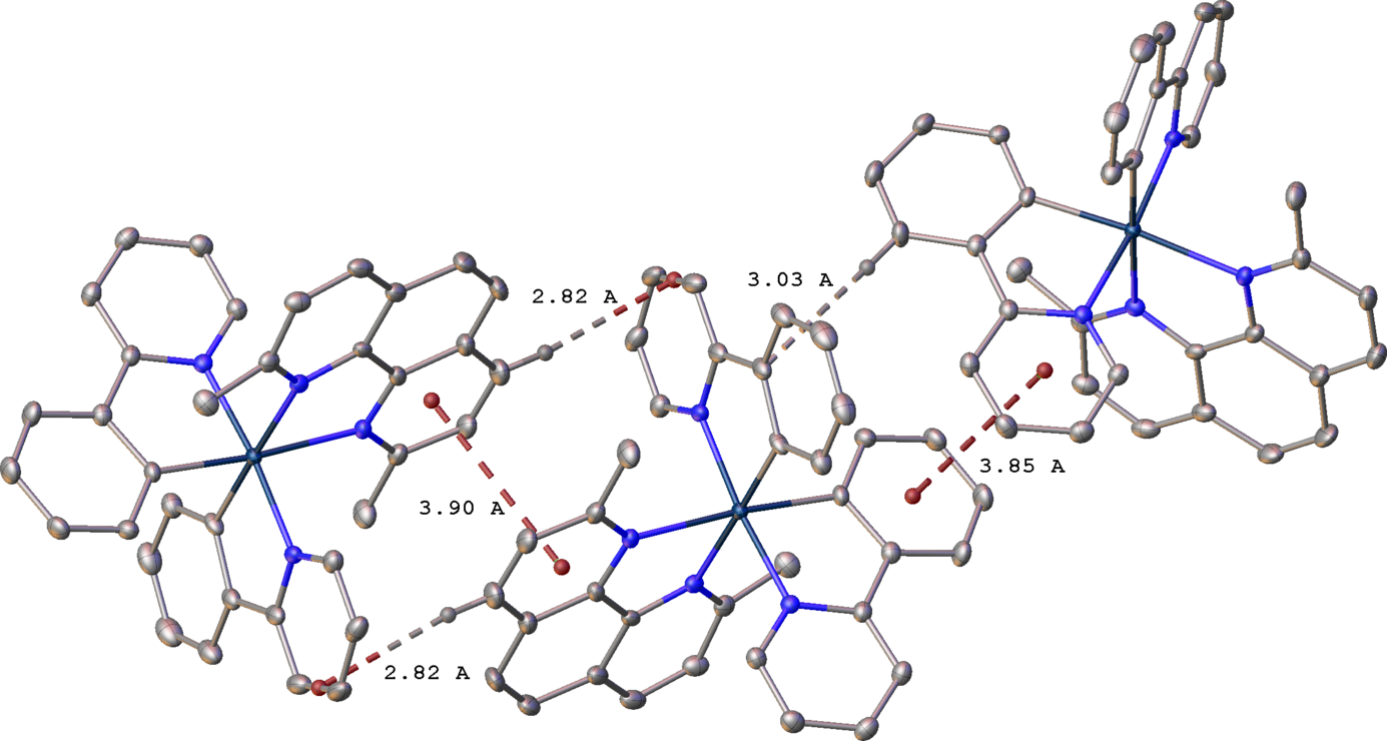
Inter­molecular π–π and C—H⋯π inter­actions between cations of **1**, related by symmetry operations −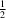 + *x*, *y*, 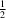 − *z* and 1 − *x*, 1 − *y*, 1 − *z*. Other H atoms are omitted.

As might be expected for mol­ecules containing multiple arene rings, there are a number of inter­molecular π–π and C—H⋯π inter­actions. In **1**, the pyridine ring N4/C29–C33 and its inversion (1 − *x*, 1 − *y*, 1 − *z*) equivalent display an offset parallel π–π inter­action, with a centroid–centroid distance of 3.903 (2) Å and a shift of 1.601 (5) Å (Fig. 3 ▸). An approximately parallel [13.83 (9)° angle between planes] offset π–π inter­action occurs between the arene ring C1–C6 and the symmetry equivalent (−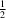 + *x*, *y*, 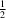 − *z*) of the pyridine ring N1/C7–C11, with a centroid–centroid distance of 3.8490 (16) Å and a shift of 1.525 (5) Å. These inter­actions continue in two dimensions, forming sheets parallel to the (010) plane. There are also C—H⋯π inter­actions between these cations at H⋯ring distances of 2.8–3.0 Å.

**Figure 4:**
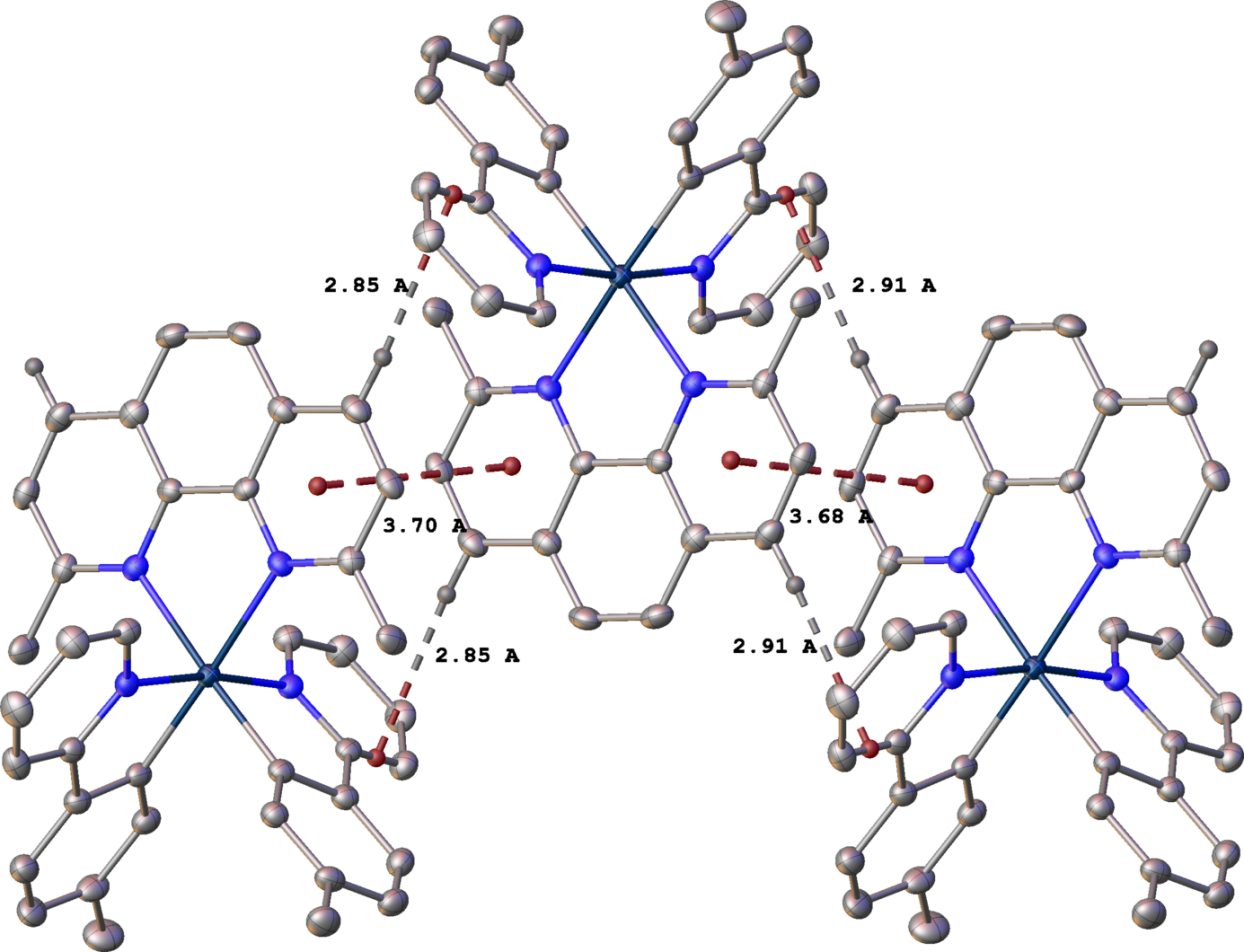
Inter­molecular π–π and C—H⋯π inter­actions between cations of **2**, related by symmetry operations 1 − *x*, 1 − *y*, 1 − *z* and 1 − *x*, −*y*, 1 − *z*. Other H atoms are omitted.

In **2**, there is also a combination of offset parallel π–π and C—H⋯π inter­actions that link the cations in one dimension along the [010] direction (Fig. 4 ▸). The pyridine ring N3/C25–C36 and its inversion (1 − *x*, 1 − *y*, 1 − *z*) equivalent have a centroid–centroid distance of 3.702 (2) Å and a shift of 1.515 (5) Å. The pyridine ring N4/C31–C35 and its inversion (1 − *x*, −*y*, 1 − *z*) equivalent have a centroid–centroid distance of 3.676 (2) Å and a shift of 1.478 (5) Å. Each pair of rings is exactly parallel due to symmetry. The C—H⋯π inter­actions are at H⋯ring distances of approximately 2.9 Å.

## 4. Database survey

A survey of the Cambridge Structural Database (CSD, version 5.45, Nov. 2023; Groom *et al.*, 2016 ▸) shows that there are 129 entries for cations containing either Ir (121) or Rh (8) with one 1,10-phenanthroline and two phenyl­pyridine ligands, without regard to substitution of the ligands. If the phenanthroline ligand is restricted to having methyl groups in the 2- and 0-positions and no additional substitutions, then the number of hits drops to two: CSD refcodes IDAKUW (Ma *et al.*, 2016 ▸) and SAWKAF (**3**, Batsanov, 2017*a* ▸). While both have the same cation as that of **1**, the former has a different counter-ion, and the latter is a deutero­chloro­form solvate. If both phenyl­pyridine ligands are restricted to having methyl groups in the five position of the phenyl ring and no additional substitutions (as in **2**), the number of hits is eight, which includes two Rh structures: CSD refcodes EFUVIM, EFUVOS (Graf *et al.*, 2014 ▸), ETUXAU (Tripathy *et al.*, 2016 ▸), GUVRAT, GUVREX (Graf *et al.*, 2020 ▸), UNEZAR (Graf, Böttcher *et al.*, 2021 ▸), XEYPOK (Graf *et al.*, 2022 ▸), and CAZVEI (Fu *et al.*, 2022 ▸).

## 5. Synthesis and crystallization

The syntheses of compounds **1** and **2** followed previously reported methods (Moon & Choe, 2013 ▸; Ma *et al.*, 2014 ▸). Orange block-shaped crystals of **1** were grown from a 5:1 mixture of di­chloro­methane and methanol layered with diethyl ether. Yellow plate-shaped crystals of **2** were obtained by vapor diffusion of diethyl ether into an aceto­nitrile solution.

## 6. Refinement

Crystal data, data collection and structure refinement details are summarized in Table 6 ▸. In **2**, the PF_6_^−^ anion was modeled as disordered over two positions with occupancies of 0.645 (6) and 0.355 (6). The disordered solvent was modeled as an overlap of a Et_2_O mol­ecule with a 0.610 (7) occupancy and two aceto­nitrile mol­ecules with 0.390 (7) occupancies. Analogous bond lengths and angles among the disordered species were restrained to be similar. Bond lengths for the aceto­nitriles mol­ecules were restrained toward ideal values. Anisotropic displacement parameters for proximal atoms were restrained to be similar.

All H atoms were placed in calculated positions with *d*(C—H) = 0.95 Å for aromatic/*sp*^2^, 0.99 Å for methyl­ene and 0.98 Å for methyl C atoms, and refined in a riding model with *U*_iso_(H) = 1.5*U*_eq_(C) for methyl H atoms and 1.2*U*_eq_(C) for the rest.

## Supplementary Material

Crystal structure: contains datablock(s) 1, 2, global. DOI: 10.1107/S2056989024012039/zv2036sup1.cif

Structure factors: contains datablock(s) 1. DOI: 10.1107/S2056989024012039/zv20361sup2.hkl

Structure factors: contains datablock(s) 2. DOI: 10.1107/S2056989024012039/zv20362sup3.hkl

CCDC references: 2409519, 2409518

Additional supporting information:  crystallographic information; 3D view; checkCIF report

## Figures and Tables

**Figure 2:**
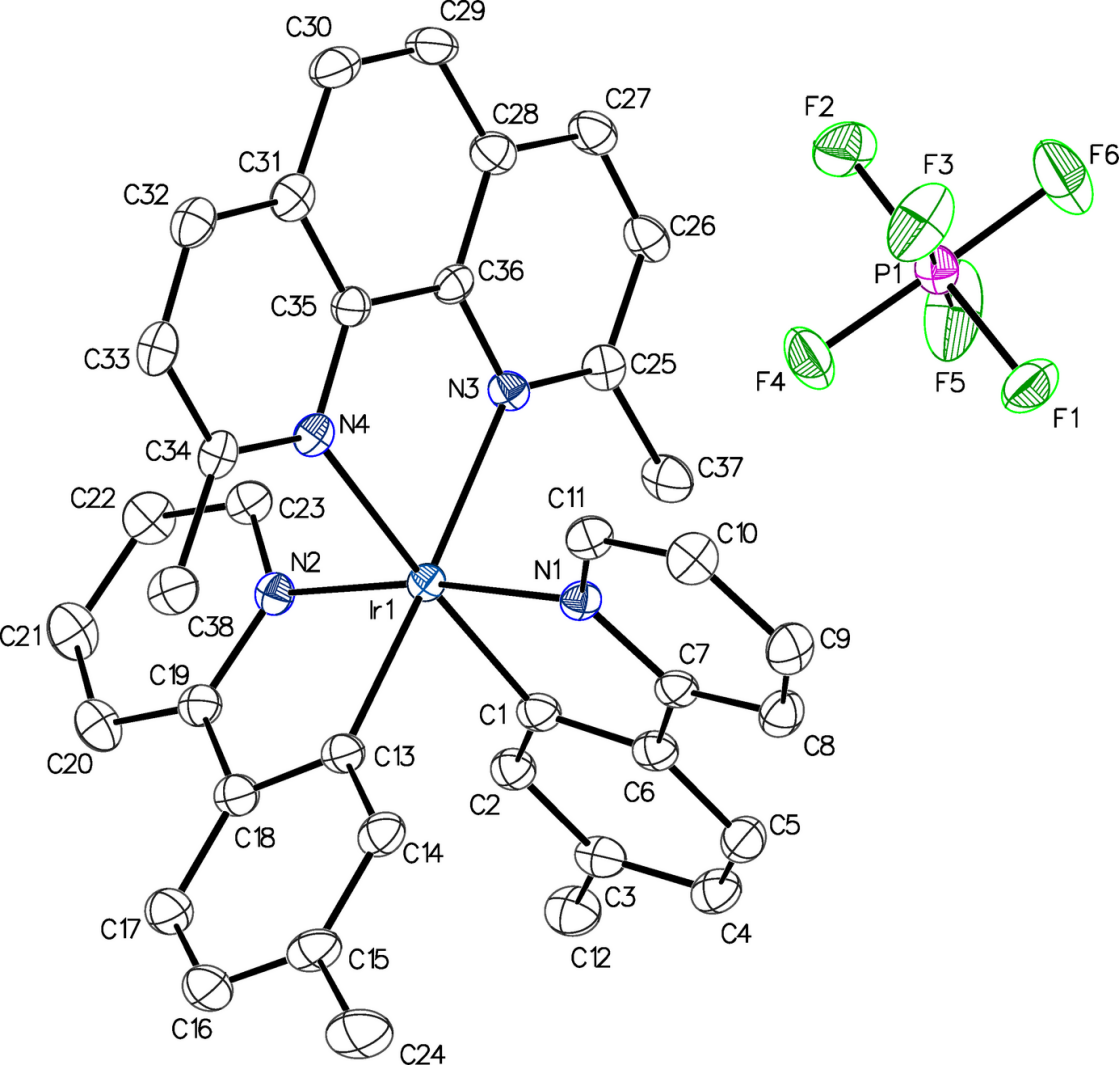
Anisotropic displacement ellipsoid plot of **2** drawn at the 50% probability level. The minor component of the anion disorder, the solvent mol­ecules and all H atoms are omitted. The PF_6_^−^ anion has been shifted with symmetry operation 1 − *x*, 1 − *y*, 1 − *z*.

**Table 1:** Selected geometric parameters (Å, °) for **1**[Chem scheme1]

Ir1—N1	2.050 (2)	Ir1—N4	2.212 (2)
Ir1—N2	2.044 (2)	Ir1—C1	2.017 (2)
Ir1—N3	2.194 (2)	Ir1—C12	2.012 (2)
			
N1—Ir1—N3	84.45 (8)	C1—Ir1—N3	99.31 (9)
N1—Ir1—N4	95.28 (8)	C1—Ir1—N4	174.88 (9)
N2—Ir1—N1	174.13 (8)	C12—Ir1—N1	96.20 (10)
N2—Ir1—N3	99.10 (8)	C12—Ir1—N2	80.36 (10)
N2—Ir1—N4	90.07 (8)	C12—Ir1—N3	178.62 (9)
N3—Ir1—N4	77.38 (8)	C12—Ir1—N4	101.33 (9)
C1—Ir1—N1	80.44 (9)	C12—Ir1—C1	82.01 (10)
C1—Ir1—N2	94.33 (9)		

**Table 2:** Selected geometric parameters (Å, °) for **2**[Chem scheme1]

Ir1—N1	2.050 (2)	Ir1—N4	2.222 (2)
Ir1—N2	2.051 (2)	Ir1—C1	2.018 (3)
Ir1—N3	2.226 (2)	Ir1—C13	2.017 (3)
			
N1—Ir1—N2	171.61 (9)	C1—Ir1—N3	101.51 (10)
N1—Ir1—N3	89.41 (9)	C1—Ir1—N4	176.54 (10)
N1—Ir1—N4	96.50 (9)	C13—Ir1—N1	93.12 (11)
N2—Ir1—N3	97.19 (9)	C13—Ir1—N2	80.39 (11)
N2—Ir1—N4	90.07 (9)	C13—Ir1—N3	177.26 (10)
N4—Ir1—N3	76.79 (9)	C13—Ir1—N4	101.85 (10)
C1—Ir1—N1	80.42 (11)	C13—Ir1—C1	79.97 (11)
C1—Ir1—N2	93.13 (11)		

**Table 3:** Selected bond lengths and angles (Å, °)

Complex	Ir—C	Ir—N_(CN)_	Ir—N_(NN)_	N_(NN)_—Ir—N_(NN)_	C—Ir—N_(NN)_	C—Ir—C
**1**	2.017 (2)	2.050 (2)	2.194 (2)	77.38 (8)	99.31 (9)	82.01 (10)
	2.012 (2)	2.044 (2)	2.212 (2)		101.33 (9)	
**2**	2.018 (3)	2.050 (2)	2.226 (2)	76.79 (9)	101.51 (10)	79.97 (11)
	2.017 (3)	2.051 (2)	2.222 (2)		101.85 (10)	
**3** ^*a*^	2.010 (3)	2.032 (3)	2.193 (3)	76.99 (10)	102.86 (12)	83.22 (13)
	2.016 (3)	2.053 (3)	2.197 (3)		97.10 (12)	
**4** ^*b*^	2.020 (3)	2.045 (3)	2.135 (2)	77.47 (9)	96.0 (1)	89.7 (1)
	2.008 (3)	2.041 (3)	2.150 (2)		96.8 (1)	

Notes: (*a*) Batsanov, 2017*a* ▸; (*b*) Batsanov, 2017*b* ▸.

**Table 4:** Hydrogen-bond geometry (Å, °) for **1**[Chem scheme1]

*D*—H⋯*A*	*D*—H	H⋯*A*	*D*⋯*A*	*D*—H⋯*A*
C10—H10⋯F1^i^	0.95	2.39	3.243 (3)	150
C11—H11⋯F3^i^	0.95	2.46	3.229 (3)	138
C22—H22⋯F1	0.95	2.45	3.311 (3)	150
C27—H27⋯F2^ii^	0.95	2.36	3.192 (3)	146
C31—H31⋯F4^iii^	0.95	2.55	3.463 (3)	160

Symmetry codes: (i) 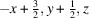; (ii) 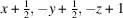; (iii) 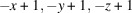.

**Table 5:** Hydrogen-bond geometry (Å, °) for **2**[Chem scheme1]

*D*—H⋯*A*	*D*—H	H⋯*A*	*D*⋯*A*	*D*—H⋯*A*
C10—H10⋯F2′	0.95	2.54	3.270 (9)	134
C10—H10⋯F2	0.95	2.53	3.329 (5)	142
C11—H11⋯F6′	0.95	2.53	3.373 (9)	149
C11—H11⋯F3	0.95	2.37	3.140 (5)	138
C22—H22⋯F6′^i^	0.95	2.51	3.272 (8)	138
C23—H23⋯F2^i^	0.95	2.32	3.194 (5)	152
C26—H26⋯F3^ii^	0.95	2.52	3.455 (5)	168
C33—H33⋯F5′^iii^	0.95	2.45	3.372 (8)	163
C37—H37*B*⋯F3′^ii^	0.98	2.37	3.330 (7)	165
C38—H38*B*⋯F5^iii^	0.98	2.42	3.393 (5)	171

Symmetry codes: (i) 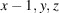; (ii) 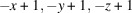; (iii) 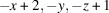.

**Table 6:** Experimental details

	**1**	**2**
Crystal data		
Chemical formula	[Ir(C_14_H_12_N_2_)(C_11_H_8_N)_2_]PF_6_	[Ir(C_14_H_12_N_2_)(C_12_H_10_N)_2_]PF_6_·0.61C_4_H_10_O·0.78C_2_H_3_N
*M*_r_	853.79	959.08
Crystal system, space group	Orthorhombic, *Pbca*	Triclinic, *P* 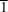
Temperature (K)	100	100
*a*, *b*, *c* (Å)	11.4130 (1), 17.1627 (1), 31.7109 (2)	9.17379 (7), 13.10065 (9), 16.55352 (14)
α, β, γ (°)	90, 90, 90	74.8888 (6), 78.9993 (7), 88.7599 (6)
*V* (Å^3^)	6211.46 (8)	1884.53 (3)
*Z*	8	2
Radiation type	Cu *K*α	Cu *K*α
μ (mm^−1^)	9.43	7.86
Crystal size (mm)	0.27 × 0.25 × 0.14	0.16 × 0.13 × 0.02

Data collection		
Diffractometer	XtaLAB Synergy, Dualflex, HyPix	XtaLAB Synergy, Dualflex, HyPix
Absorption correction	Multi-scan (*CrysAlis PRO*; Rigaku OD, 2023 ▸)	Multi-scan (*CrysAlis PRO*; Rigaku OD, 2023 ▸)
*T*_min_, *T*_max_	0.558, 1.000	0.509, 1.000
No. of measured, independent and observed [*I* > 2σ(*I*)] reflections	101540, 6757, 6668	62414, 8074, 7770
*R*_int_	0.042	0.058
(sin θ/λ)_max_ (Å^−1^)	0.640	0.639

Refinement		
*R*[*F*^2^ > 2σ(*F*^2^)], *wR*(*F*^2^), *S*	0.024, 0.060, 1.16	0.026, 0.067, 1.04
No. of reflections	6757	8074
No. of parameters	436	624
No. of restraints	0	441
H-atom treatment	H-atom parameters constrained	H-atom parameters constrained
Δρ_max_, Δρ_min_ (e Å^−3^)	1.12, −0.68	1.25, −1.04

Computer programs: *CrysAlis PRO* (Rigaku OD, 2023 ▸), *SHELXT2018/2* (Sheldrick, 2015*a* ▸), *SHELXL2019/3* (Sheldrick, 2015*b* ▸) and *OLEX2* (Dolomanov *et al.*, 2009 ▸).

## supplementary crystallographic information

### (2,9-Dimethyl-1,10-phenanthroline)bis[2-(pyridin-2-yl)phenyl]iridium(III) hexafluorophosphate (1) Crystal data

**Table:**

[Ir(C_14_H_12_N_2_)(C_11_H_8_N)_2_]PF_6_	*D*_x_ = 1.826 Mg m^−^^3^
*M**_r_* = 853.79	Cu *K*α radiation, λ = 1.54184 Å
Orthorhombic, *P**b**c**a*	Cell parameters from 61784 reflections
*a* = 11.4130 (1) Å	θ = 4.8–80.1°
*b* = 17.1627 (1) Å	µ = 9.43 mm^−^^1^
*c* = 31.7109 (2) Å	*T* = 100 K
*V* = 6211.46 (8) Å^3^	Block, orange
*Z* = 8	0.27 × 0.25 × 0.14 mm
*F*(000) = 3344	

### (2,9-Dimethyl-1,10-phenanthroline)bis[2-(pyridin-2-yl)phenyl]iridium(III) hexafluorophosphate (1) Data collection

**Table:**

XtaLAB Synergy, Dualflex, HyPix diffractometer	6757 independent reflections
Radiation source: micro-focus sealed X-ray tube, PhotonJet (Cu) X-ray Source	6668 reflections with *I* > 2σ(*I*)
Mirror monochromator	*R*_int_ = 0.042
Detector resolution: 10.0000 pixels mm^-1^	θ_max_ = 80.5°, θ_min_ = 4.8°
ω scans	*h* = −14→14
Absorption correction: multi-scan (CrysAlisPro; Rigaku OD, 2023)	*k* = −21→20
*T*_min_ = 0.558, *T*_max_ = 1.000	*l* = −39→40
101540 measured reflections	

### (2,9-Dimethyl-1,10-phenanthroline)bis[2-(pyridin-2-yl)phenyl]iridium(III) hexafluorophosphate (1) Refinement

**Table:**

Refinement on *F*^2^	Secondary atom site location: difference Fourier map
Least-squares matrix: full	Hydrogen site location: inferred from neighbouring sites
*R*[*F*^2^ > 2σ(*F*^2^)] = 0.024	H-atom parameters constrained
*wR*(*F*^2^) = 0.060	*w* = 1/[σ^2^(*F*_o_^2^) + (0.0229*P*)^2^ + 11.8694*P*] where *P* = (*F*_o_^2^ + 2*F*_c_^2^)/3
*S* = 1.16	(Δ/σ)_max_ = 0.004
6757 reflections	Δρ_max_ = 1.12 e Å^−^^3^
436 parameters	Δρ_min_ = −0.68 e Å^−^^3^
0 restraints	Extinction correction: *SHELXL2019/3* (Sheldrick, 2015b), Fc^*^=kFc[1+0.001xFc^2^λ^3^/sin(2θ)]^-1/4^
Primary atom site location: dual	Extinction coefficient: 0.000151 (6)

### (2,9-Dimethyl-1,10-phenanthroline)bis[2-(pyridin-2-yl)phenyl]iridium(III) hexafluorophosphate (1) Special details

**Table:**

Geometry. All esds (except the esd in the dihedral angle between two l.s. planes) are estimated using the full covariance matrix. The cell esds are taken into account individually in the estimation of esds in distances, angles and torsion angles; correlations between esds in cell parameters are only used when they are defined by crystal symmetry. An approximate (isotropic) treatment of cell esds is used for estimating esds involving l.s. planes.

### (2,9-Dimethyl-1,10-phenanthroline)bis[2-(pyridin-2-yl)phenyl]iridium(III) hexafluorophosphate (1) Fractional atomic coordinates and isotropic or equivalent isotropic displacement parameters (Å^2^)

**Table:**

	*x*	*y*	*z*	*U*_iso_*/*U*_eq_	
Ir1	0.51064 (2)	0.49405 (2)	0.35682 (2)	0.01242 (5)	
N1	0.64998 (18)	0.54667 (12)	0.32760 (6)	0.0139 (4)	
N2	0.36244 (18)	0.44319 (13)	0.38033 (6)	0.0159 (4)	
N3	0.63668 (18)	0.40108 (12)	0.37084 (6)	0.0146 (4)	
N4	0.56784 (19)	0.52297 (13)	0.42162 (6)	0.0162 (4)	
C1	0.4738 (2)	0.46697 (15)	0.29637 (7)	0.0152 (5)	
C2	0.3806 (2)	0.42257 (15)	0.28099 (8)	0.0180 (5)	
H2	0.320529	0.406073	0.299715	0.022*	
C3	0.3746 (2)	0.40217 (16)	0.23856 (8)	0.0215 (5)	
H3	0.311915	0.370417	0.228920	0.026*	
C4	0.4589 (3)	0.42752 (16)	0.21007 (8)	0.0229 (5)	
H4	0.454588	0.412515	0.181280	0.027*	
C5	0.5493 (2)	0.47488 (16)	0.22401 (8)	0.0208 (5)	
H5	0.606228	0.493618	0.204648	0.025*	
C6	0.5565 (2)	0.49509 (13)	0.26687 (8)	0.0160 (5)	
C7	0.6527 (2)	0.54057 (14)	0.28459 (8)	0.0157 (5)	
C8	0.7438 (2)	0.57419 (15)	0.26173 (8)	0.0187 (5)	
H8	0.743229	0.572399	0.231788	0.022*	
C9	0.8353 (2)	0.61024 (15)	0.28279 (8)	0.0200 (5)	
H9	0.898527	0.632459	0.267446	0.024*	
C10	0.8337 (2)	0.61356 (15)	0.32644 (8)	0.0201 (5)	
H10	0.896565	0.636951	0.341467	0.024*	
C11	0.7391 (2)	0.58224 (15)	0.34773 (8)	0.0170 (5)	
H11	0.736895	0.585921	0.377606	0.020*	
C12	0.3946 (2)	0.57973 (15)	0.34547 (8)	0.0175 (5)	
C13	0.4154 (2)	0.65210 (16)	0.32684 (8)	0.0210 (5)	
H13	0.492169	0.664552	0.317475	0.025*	
C14	0.3253 (3)	0.70679 (17)	0.32166 (9)	0.0278 (6)	
H14	0.341283	0.755710	0.308878	0.033*	
C15	0.2124 (3)	0.68921 (18)	0.33530 (9)	0.0301 (7)	
H15	0.151613	0.726569	0.332250	0.036*	
C16	0.1886 (3)	0.61752 (18)	0.35329 (8)	0.0251 (6)	
H16	0.111499	0.605614	0.362596	0.030*	
C17	0.2786 (2)	0.56242 (16)	0.35773 (7)	0.0190 (5)	
C18	0.2621 (2)	0.48607 (16)	0.37700 (8)	0.0190 (5)	
C19	0.1564 (2)	0.45492 (18)	0.39137 (8)	0.0253 (6)	
H19	0.086445	0.484707	0.389581	0.030*	
C20	0.1538 (2)	0.38064 (19)	0.40823 (9)	0.0275 (6)	
H20	0.081877	0.359062	0.417796	0.033*	
C21	0.2567 (3)	0.33762 (17)	0.41112 (8)	0.0244 (6)	
H21	0.256365	0.286435	0.422525	0.029*	
C22	0.3596 (2)	0.37113 (15)	0.39702 (8)	0.0195 (5)	
H22	0.430497	0.342410	0.399172	0.023*	
C23	0.6701 (2)	0.34073 (14)	0.34655 (8)	0.0172 (5)	
C24	0.7709 (2)	0.29651 (16)	0.35595 (8)	0.0208 (5)	
H24	0.795756	0.256781	0.337108	0.025*	
C25	0.8328 (2)	0.31040 (16)	0.39189 (9)	0.0227 (5)	
H25	0.900264	0.280176	0.398306	0.027*	
C26	0.7964 (2)	0.36956 (16)	0.41930 (8)	0.0210 (5)	
C27	0.8525 (2)	0.38278 (19)	0.45890 (9)	0.0274 (6)	
H27	0.917596	0.351582	0.466879	0.033*	
C28	0.8140 (3)	0.4390 (2)	0.48504 (9)	0.0299 (7)	
H28	0.851634	0.446614	0.511451	0.036*	
C29	0.7176 (2)	0.48733 (17)	0.47356 (8)	0.0229 (6)	
C30	0.6761 (3)	0.54628 (19)	0.50012 (8)	0.0270 (6)	
H30	0.712731	0.555431	0.526566	0.032*	
C31	0.5832 (2)	0.59043 (17)	0.48793 (8)	0.0233 (5)	
H31	0.553931	0.629698	0.506237	0.028*	
C32	0.5299 (2)	0.57849 (16)	0.44842 (8)	0.0194 (5)	
C33	0.6595 (2)	0.47651 (16)	0.43452 (8)	0.0179 (5)	
C34	0.6989 (2)	0.41516 (15)	0.40718 (8)	0.0167 (5)	
C35	0.5974 (2)	0.31599 (15)	0.30974 (9)	0.0221 (5)	
H35A	0.514957	0.312952	0.318246	0.033*	
H35B	0.623651	0.264746	0.299921	0.033*	
H35C	0.605828	0.354052	0.286903	0.033*	
C36	0.4290 (3)	0.63030 (17)	0.43709 (9)	0.0258 (6)	
H36A	0.447145	0.658624	0.411067	0.039*	
H36B	0.415152	0.667579	0.459963	0.039*	
H36C	0.358563	0.598554	0.432826	0.039*	
P1	0.56252 (6)	0.18708 (4)	0.44462 (2)	0.02319 (15)	
F1	0.53641 (16)	0.21821 (10)	0.39759 (5)	0.0289 (4)	
F2	0.58804 (17)	0.15683 (15)	0.49122 (6)	0.0459 (5)	
F3	0.69740 (15)	0.17593 (10)	0.43303 (5)	0.0294 (4)	
F4	0.58775 (18)	0.27491 (12)	0.45889 (6)	0.0415 (5)	
F5	0.42699 (16)	0.19877 (14)	0.45578 (6)	0.0463 (5)	
F6	0.5363 (2)	0.10019 (11)	0.42921 (8)	0.0502 (6)	

### (2,9-Dimethyl-1,10-phenanthroline)bis[2-(pyridin-2-yl)phenyl]iridium(III) hexafluorophosphate (1) Atomic displacement parameters (Å^2^)

**Table:**

	*U*^11^	*U*^22^	*U*^33^	*U*^12^	*U*^13^	*U*^23^
Ir1	0.01142 (7)	0.01540 (7)	0.01044 (7)	0.00033 (3)	−0.00051 (3)	−0.00022 (3)
N1	0.0134 (9)	0.0143 (9)	0.0139 (9)	0.0014 (7)	0.0006 (7)	−0.0008 (7)
N2	0.0120 (9)	0.0239 (11)	0.0118 (9)	−0.0019 (8)	−0.0007 (7)	−0.0020 (8)
N3	0.0134 (9)	0.0158 (10)	0.0147 (10)	−0.0004 (8)	0.0010 (8)	0.0031 (8)
N4	0.0146 (10)	0.0222 (10)	0.0118 (9)	−0.0024 (8)	−0.0004 (8)	0.0003 (8)
C1	0.0164 (11)	0.0182 (12)	0.0109 (11)	0.0027 (9)	−0.0016 (9)	−0.0009 (9)
C2	0.0144 (11)	0.0220 (12)	0.0175 (12)	0.0006 (9)	−0.0006 (9)	−0.0021 (10)
C3	0.0192 (12)	0.0261 (13)	0.0193 (13)	−0.0007 (10)	−0.0048 (10)	−0.0052 (10)
C4	0.0274 (14)	0.0266 (13)	0.0146 (12)	0.0004 (11)	−0.0035 (10)	−0.0050 (10)
C5	0.0252 (13)	0.0243 (13)	0.0130 (12)	−0.0011 (11)	0.0020 (10)	−0.0029 (10)
C6	0.0181 (13)	0.0146 (11)	0.0152 (12)	0.0021 (9)	−0.0004 (10)	−0.0003 (8)
C7	0.0169 (11)	0.0164 (11)	0.0140 (11)	0.0024 (9)	0.0005 (9)	0.0004 (9)
C8	0.0228 (12)	0.0183 (12)	0.0150 (11)	−0.0008 (10)	0.0018 (10)	−0.0005 (9)
C9	0.0216 (13)	0.0176 (12)	0.0209 (12)	−0.0020 (10)	0.0053 (10)	−0.0010 (10)
C10	0.0190 (12)	0.0181 (12)	0.0231 (13)	−0.0022 (10)	−0.0018 (10)	0.0003 (10)
C11	0.0185 (12)	0.0166 (12)	0.0160 (11)	0.0003 (9)	−0.0018 (10)	−0.0002 (9)
C12	0.0181 (12)	0.0200 (12)	0.0145 (11)	0.0087 (10)	−0.0044 (9)	−0.0038 (9)
C13	0.0238 (13)	0.0218 (13)	0.0176 (12)	0.0043 (10)	−0.0026 (10)	−0.0007 (10)
C14	0.0379 (16)	0.0220 (13)	0.0235 (13)	0.0091 (12)	−0.0062 (12)	−0.0017 (11)
C15	0.0314 (15)	0.0313 (15)	0.0275 (15)	0.0146 (12)	−0.0060 (12)	−0.0081 (12)
C16	0.0216 (14)	0.0331 (15)	0.0206 (13)	0.0089 (12)	−0.0036 (10)	−0.0076 (11)
C17	0.0182 (13)	0.0261 (14)	0.0128 (11)	0.0049 (10)	−0.0024 (9)	−0.0055 (9)
C18	0.0154 (12)	0.0294 (14)	0.0121 (11)	0.0028 (10)	−0.0009 (9)	−0.0067 (10)
C19	0.0157 (12)	0.0396 (16)	0.0205 (13)	−0.0008 (11)	0.0003 (10)	−0.0081 (11)
C20	0.0169 (13)	0.0457 (17)	0.0199 (13)	−0.0104 (12)	0.0022 (10)	−0.0047 (12)
C21	0.0267 (14)	0.0282 (14)	0.0181 (12)	−0.0081 (11)	0.0046 (11)	−0.0022 (10)
C22	0.0204 (12)	0.0226 (13)	0.0154 (12)	−0.0022 (10)	−0.0003 (10)	−0.0014 (9)
C23	0.0179 (12)	0.0158 (11)	0.0180 (11)	−0.0011 (9)	0.0017 (10)	0.0043 (9)
C24	0.0174 (12)	0.0174 (12)	0.0275 (14)	0.0008 (10)	0.0021 (10)	0.0024 (10)
C25	0.0144 (12)	0.0247 (13)	0.0291 (14)	0.0001 (10)	0.0000 (10)	0.0089 (11)
C26	0.0145 (12)	0.0287 (14)	0.0197 (12)	−0.0004 (10)	−0.0002 (10)	0.0078 (10)
C27	0.0159 (12)	0.0434 (17)	0.0229 (14)	0.0043 (12)	−0.0032 (10)	0.0086 (12)
C28	0.0217 (14)	0.0507 (19)	0.0175 (13)	0.0022 (13)	−0.0072 (11)	0.0046 (12)
C29	0.0192 (13)	0.0350 (15)	0.0143 (12)	−0.0020 (11)	−0.0019 (10)	0.0022 (10)
C30	0.0265 (14)	0.0389 (17)	0.0155 (13)	−0.0024 (13)	−0.0041 (10)	−0.0029 (11)
C31	0.0242 (13)	0.0312 (14)	0.0145 (12)	−0.0029 (11)	0.0007 (10)	−0.0049 (10)
C32	0.0191 (12)	0.0249 (13)	0.0142 (11)	−0.0039 (10)	0.0018 (10)	−0.0014 (10)
C33	0.0147 (11)	0.0258 (13)	0.0131 (11)	−0.0025 (10)	−0.0016 (9)	0.0032 (10)
C34	0.0102 (11)	0.0237 (12)	0.0160 (11)	−0.0018 (9)	−0.0004 (9)	0.0052 (9)
C35	0.0252 (13)	0.0159 (12)	0.0253 (13)	0.0000 (10)	−0.0033 (11)	−0.0024 (10)
C36	0.0312 (15)	0.0284 (14)	0.0180 (13)	0.0064 (12)	−0.0014 (11)	−0.0069 (11)
P1	0.0207 (3)	0.0297 (4)	0.0191 (3)	−0.0029 (3)	0.0029 (3)	0.0051 (3)
F1	0.0373 (9)	0.0296 (9)	0.0199 (8)	0.0045 (8)	0.0034 (7)	0.0042 (7)
F2	0.0260 (9)	0.0885 (17)	0.0234 (9)	0.0076 (10)	0.0055 (7)	0.0220 (10)
F3	0.0265 (8)	0.0344 (9)	0.0273 (8)	0.0024 (7)	0.0088 (7)	0.0029 (7)
F4	0.0410 (11)	0.0407 (11)	0.0429 (11)	0.0032 (9)	0.0030 (9)	−0.0189 (9)
F5	0.0208 (9)	0.0781 (15)	0.0399 (11)	0.0010 (9)	0.0053 (8)	0.0287 (10)
F6	0.0563 (13)	0.0248 (10)	0.0696 (15)	−0.0129 (9)	−0.0256 (12)	0.0134 (10)

### (2,9-Dimethyl-1,10-phenanthroline)bis[2-(pyridin-2-yl)phenyl]iridium(III) hexafluorophosphate (1) Geometric parameters (Å, º)

**Table:**

Ir1—N1	2.050 (2)	C16—C17	1.404 (4)
Ir1—N2	2.044 (2)	C17—C18	1.458 (4)
Ir1—N3	2.194 (2)	C18—C19	1.396 (4)
Ir1—N4	2.212 (2)	C19—H19	0.9500
Ir1—C1	2.017 (2)	C19—C20	1.383 (4)
Ir1—C12	2.012 (2)	C20—H20	0.9500
N1—C7	1.368 (3)	C20—C21	1.391 (4)
N1—C11	1.347 (3)	C21—H21	0.9500
N2—C18	1.365 (3)	C21—C22	1.381 (4)
N2—C22	1.346 (3)	C22—H22	0.9500
N3—C23	1.346 (3)	C23—C24	1.410 (4)
N3—C34	1.375 (3)	C23—C35	1.494 (4)
N4—C32	1.348 (3)	C24—H24	0.9500
N4—C33	1.378 (3)	C24—C25	1.362 (4)
C1—C2	1.396 (3)	C25—H25	0.9500
C1—C6	1.414 (4)	C25—C26	1.400 (4)
C2—H2	0.9500	C26—C27	1.428 (4)
C2—C3	1.392 (4)	C26—C34	1.413 (3)
C3—H3	0.9500	C27—H27	0.9500
C3—C4	1.389 (4)	C27—C28	1.346 (4)
C4—H4	0.9500	C28—H28	0.9500
C4—C5	1.386 (4)	C28—C29	1.426 (4)
C5—H5	0.9500	C29—C30	1.399 (4)
C5—C6	1.405 (3)	C29—C33	1.416 (4)
C6—C7	1.460 (4)	C30—H30	0.9500
C7—C8	1.393 (4)	C30—C31	1.360 (4)
C8—H8	0.9500	C31—H31	0.9500
C8—C9	1.385 (4)	C31—C32	1.408 (4)
C9—H9	0.9500	C32—C36	1.499 (4)
C9—C10	1.385 (4)	C33—C34	1.436 (4)
C10—H10	0.9500	C35—H35A	0.9800
C10—C11	1.382 (4)	C35—H35B	0.9800
C11—H11	0.9500	C35—H35C	0.9800
C12—C13	1.396 (4)	C36—H36A	0.9800
C12—C17	1.411 (4)	C36—H36B	0.9800
C13—H13	0.9500	C36—H36C	0.9800
C13—C14	1.401 (4)	P1—F1	1.6118 (17)
C14—H14	0.9500	P1—F2	1.5932 (19)
C14—C15	1.392 (5)	P1—F3	1.5943 (18)
C15—H15	0.9500	P1—F4	1.600 (2)
C15—C16	1.383 (5)	P1—F5	1.599 (2)
C16—H16	0.9500	P1—F6	1.597 (2)
			
N1—Ir1—N3	84.45 (8)	N2—C18—C17	114.1 (2)
N1—Ir1—N4	95.28 (8)	N2—C18—C19	119.5 (3)
N2—Ir1—N1	174.13 (8)	C19—C18—C17	126.3 (3)
N2—Ir1—N3	99.10 (8)	C18—C19—H19	120.1
N2—Ir1—N4	90.07 (8)	C20—C19—C18	119.9 (3)
N3—Ir1—N4	77.38 (8)	C20—C19—H19	120.1
C1—Ir1—N1	80.44 (9)	C19—C20—H20	120.1
C1—Ir1—N2	94.33 (9)	C19—C20—C21	119.8 (3)
C1—Ir1—N3	99.31 (9)	C21—C20—H20	120.1
C1—Ir1—N4	174.88 (9)	C20—C21—H21	120.8
C12—Ir1—N1	96.20 (10)	C22—C21—C20	118.4 (3)
C12—Ir1—N2	80.36 (10)	C22—C21—H21	120.8
C12—Ir1—N3	178.62 (9)	N2—C22—C21	122.0 (3)
C12—Ir1—N4	101.33 (9)	N2—C22—H22	119.0
C12—Ir1—C1	82.01 (10)	C21—C22—H22	119.0
C7—N1—Ir1	115.75 (16)	N3—C23—C24	121.6 (2)
C11—N1—Ir1	124.85 (17)	N3—C23—C35	120.6 (2)
C11—N1—C7	119.3 (2)	C24—C23—C35	117.7 (2)
C18—N2—Ir1	115.82 (18)	C23—C24—H24	119.8
C22—N2—Ir1	123.77 (18)	C25—C24—C23	120.4 (3)
C22—N2—C18	120.4 (2)	C25—C24—H24	119.8
C23—N3—Ir1	129.02 (17)	C24—C25—H25	120.3
C23—N3—C34	117.9 (2)	C24—C25—C26	119.5 (2)
C34—N3—Ir1	112.38 (16)	C26—C25—H25	120.3
C32—N4—Ir1	130.48 (18)	C25—C26—C27	121.9 (3)
C32—N4—C33	117.8 (2)	C25—C26—C34	117.8 (2)
C33—N4—Ir1	111.72 (16)	C34—C26—C27	120.3 (3)
C2—C1—Ir1	128.08 (19)	C26—C27—H27	119.7
C2—C1—C6	117.6 (2)	C28—C27—C26	120.6 (3)
C6—C1—Ir1	114.25 (18)	C28—C27—H27	119.7
C1—C2—H2	119.6	C27—C28—H28	119.6
C3—C2—C1	120.8 (2)	C27—C28—C29	120.8 (3)
C3—C2—H2	119.6	C29—C28—H28	119.6
C2—C3—H3	119.5	C30—C29—C28	121.9 (3)
C4—C3—C2	121.0 (2)	C30—C29—C33	117.6 (3)
C4—C3—H3	119.5	C33—C29—C28	120.5 (3)
C3—C4—H4	120.3	C29—C30—H30	120.1
C5—C4—C3	119.5 (2)	C31—C30—C29	119.7 (2)
C5—C4—H4	120.3	C31—C30—H30	120.1
C4—C5—H5	120.1	C30—C31—H31	119.7
C4—C5—C6	119.8 (2)	C30—C31—C32	120.6 (3)
C6—C5—H5	120.1	C32—C31—H31	119.7
C1—C6—C7	115.5 (2)	N4—C32—C31	121.7 (3)
C5—C6—C1	121.1 (2)	N4—C32—C36	121.0 (2)
C5—C6—C7	123.2 (2)	C31—C32—C36	117.3 (2)
N1—C7—C6	114.0 (2)	N4—C33—C29	122.6 (2)
N1—C7—C8	120.3 (2)	N4—C33—C34	118.9 (2)
C8—C7—C6	125.7 (2)	C29—C33—C34	118.5 (2)
C7—C8—H8	120.1	N3—C34—C26	122.5 (2)
C9—C8—C7	119.8 (2)	N3—C34—C33	118.2 (2)
C9—C8—H8	120.1	C26—C34—C33	119.3 (2)
C8—C9—H9	120.3	C23—C35—H35A	109.5
C10—C9—C8	119.3 (2)	C23—C35—H35B	109.5
C10—C9—H9	120.3	C23—C35—H35C	109.5
C9—C10—H10	120.6	H35A—C35—H35B	109.5
C11—C10—C9	118.8 (2)	H35A—C35—H35C	109.5
C11—C10—H10	120.6	H35B—C35—H35C	109.5
N1—C11—C10	122.3 (2)	C32—C36—H36A	109.5
N1—C11—H11	118.8	C32—C36—H36B	109.5
C10—C11—H11	118.8	C32—C36—H36C	109.5
C13—C12—Ir1	127.9 (2)	H36A—C36—H36B	109.5
C13—C12—C17	117.6 (2)	H36A—C36—H36C	109.5
C17—C12—Ir1	114.48 (19)	H36B—C36—H36C	109.5
C12—C13—H13	119.3	F2—P1—F1	179.64 (13)
C12—C13—C14	121.4 (3)	F2—P1—F3	89.89 (10)
C14—C13—H13	119.3	F2—P1—F4	90.67 (13)
C13—C14—H14	120.1	F2—P1—F5	90.71 (10)
C15—C14—C13	119.8 (3)	F2—P1—F6	90.78 (13)
C15—C14—H14	120.1	F3—P1—F1	90.29 (9)
C14—C15—H15	119.9	F3—P1—F4	90.26 (10)
C16—C15—C14	120.2 (3)	F3—P1—F5	179.38 (11)
C16—C15—H15	119.9	F3—P1—F6	89.88 (12)
C15—C16—H16	120.1	F4—P1—F1	89.01 (11)
C15—C16—C17	119.8 (3)	F5—P1—F1	89.11 (10)
C17—C16—H16	120.1	F5—P1—F4	89.62 (12)
C12—C17—C18	115.2 (2)	F6—P1—F1	89.53 (11)
C16—C17—C12	121.1 (3)	F6—P1—F4	178.54 (12)
C16—C17—C18	123.6 (3)	F6—P1—F5	90.23 (13)
			
Ir1—N1—C7—C6	−1.7 (3)	C13—C12—C17—C18	180.0 (2)
Ir1—N1—C7—C8	−179.89 (18)	C13—C14—C15—C16	−1.1 (4)
Ir1—N1—C11—C10	176.85 (19)	C14—C15—C16—C17	0.0 (4)
Ir1—N2—C18—C17	−1.2 (3)	C15—C16—C17—C12	2.2 (4)
Ir1—N2—C18—C19	178.51 (18)	C15—C16—C17—C18	178.8 (2)
Ir1—N2—C22—C21	−177.47 (19)	C16—C17—C18—N2	−176.5 (2)
Ir1—N3—C23—C24	165.04 (18)	C16—C17—C18—C19	3.8 (4)
Ir1—N3—C23—C35	−18.1 (3)	C17—C12—C13—C14	2.0 (4)
Ir1—N3—C34—C26	−170.57 (19)	C17—C18—C19—C20	178.6 (2)
Ir1—N3—C34—C33	12.2 (3)	C18—N2—C22—C21	0.3 (4)
Ir1—N4—C32—C31	−176.17 (19)	C18—C19—C20—C21	0.6 (4)
Ir1—N4—C32—C36	3.6 (4)	C19—C20—C21—C22	0.2 (4)
Ir1—N4—C33—C29	175.2 (2)	C20—C21—C22—N2	−0.7 (4)
Ir1—N4—C33—C34	−4.1 (3)	C22—N2—C18—C17	−179.1 (2)
Ir1—C1—C2—C3	−173.0 (2)	C22—N2—C18—C19	0.6 (4)
Ir1—C1—C6—C5	173.9 (2)	C23—N3—C34—C26	0.9 (3)
Ir1—C1—C6—C7	−1.6 (3)	C23—N3—C34—C33	−176.3 (2)
Ir1—C12—C13—C14	−178.8 (2)	C23—C24—C25—C26	−0.6 (4)
Ir1—C12—C17—C16	177.60 (19)	C24—C25—C26—C27	175.1 (3)
Ir1—C12—C17—C18	0.7 (3)	C24—C25—C26—C34	−3.0 (4)
N1—C7—C8—C9	3.5 (4)	C25—C26—C27—C28	−178.6 (3)
N2—C18—C19—C20	−1.0 (4)	C25—C26—C34—N3	3.0 (4)
N3—C23—C24—C25	4.8 (4)	C25—C26—C34—C33	−179.8 (2)
N4—C33—C34—N3	−5.6 (3)	C26—C27—C28—C29	−0.9 (5)
N4—C33—C34—C26	177.1 (2)	C27—C26—C34—N3	−175.2 (2)
C1—C2—C3—C4	−2.2 (4)	C27—C26—C34—C33	2.0 (4)
C1—C6—C7—N1	2.2 (3)	C27—C28—C29—C30	−179.9 (3)
C1—C6—C7—C8	−179.8 (2)	C27—C28—C29—C33	0.6 (5)
C2—C1—C6—C5	−4.0 (4)	C28—C29—C30—C31	−179.5 (3)
C2—C1—C6—C7	−179.5 (2)	C28—C29—C33—N4	−178.3 (3)
C2—C3—C4—C5	−1.0 (4)	C28—C29—C33—C34	0.9 (4)
C3—C4—C5—C6	1.6 (4)	C29—C30—C31—C32	−1.4 (4)
C4—C5—C6—C1	0.9 (4)	C29—C33—C34—N3	175.1 (2)
C4—C5—C6—C7	176.0 (2)	C29—C33—C34—C26	−2.2 (4)
C5—C6—C7—N1	−173.2 (2)	C30—C29—C33—N4	2.1 (4)
C5—C6—C7—C8	4.8 (4)	C30—C29—C33—C34	−178.6 (3)
C6—C1—C2—C3	4.6 (4)	C30—C31—C32—N4	0.6 (4)
C6—C7—C8—C9	−174.4 (2)	C30—C31—C32—C36	−179.2 (3)
C7—N1—C11—C10	0.4 (4)	C32—N4—C33—C29	−2.9 (4)
C7—C8—C9—C10	−1.2 (4)	C32—N4—C33—C34	177.8 (2)
C8—C9—C10—C11	−1.5 (4)	C33—N4—C32—C31	1.5 (4)
C9—C10—C11—N1	1.9 (4)	C33—N4—C32—C36	−178.7 (2)
C11—N1—C7—C6	175.0 (2)	C33—C29—C30—C31	0.1 (4)
C11—N1—C7—C8	−3.2 (4)	C34—N3—C23—C24	−4.8 (3)
C12—C13—C14—C15	0.1 (4)	C34—N3—C23—C35	172.1 (2)
C12—C17—C18—N2	0.3 (3)	C34—C26—C27—C28	−0.5 (4)
C12—C17—C18—C19	−179.4 (2)	C35—C23—C24—C25	−172.2 (2)
C13—C12—C17—C16	−3.1 (4)		

### (2,9-Dimethyl-1,10-phenanthroline)bis[2-(pyridin-2-yl)phenyl]iridium(III) hexafluorophosphate (1) Hydrogen-bond geometry (Å, º)

**Table:**

*D*—H···*A*	*D*—H	H···*A*	*D*···*A*	*D*—H···*A*
C10—H10···F1^i^	0.95	2.39	3.243 (3)	150
C11—H11···F3^i^	0.95	2.46	3.229 (3)	138
C22—H22···F1	0.95	2.45	3.311 (3)	150
C27—H27···F2^ii^	0.95	2.36	3.192 (3)	146
C31—H31···F4^iii^	0.95	2.55	3.463 (3)	160

Symmetry codes: (i) −*x*+3/2, *y*+1/2, *z*; (ii) *x*+1/2, −*y*+1/2, −*z*+1; (iii) −*x*+1, −*y*+1, −*z*+1.

### (2,9-Dimethyl-1,10-phenanthroline)bis[5-methyl-2-(pyridin-2-yl)phenyl]iridium(III) hexafluorophosphate–diethyl ether–acetonitrile (1/0.61/0.78) (2) Crystal data

**Table:**

[Ir(C_14_H_12_N_2_)(C_12_H_10_N)_2_]PF_6_·0.61C_4_H_10_O·0.78C_2_H_3_N	*Z* = 2
*M**_r_* = 959.08	*F*(000) = 954
Triclinic, *P*1	*D*_x_ = 1.690 Mg m^−^^3^
*a* = 9.17379 (7) Å	Cu *K*α radiation, λ = 1.54184 Å
*b* = 13.10065 (9) Å	Cell parameters from 40596 reflections
*c* = 16.55352 (14) Å	θ = 2.8–79.7°
α = 74.8888 (6)°	µ = 7.86 mm^−^^1^
β = 78.9993 (7)°	*T* = 100 K
γ = 88.7599 (6)°	Plate, yellow
*V* = 1884.53 (3) Å^3^	0.16 × 0.13 × 0.02 mm

### (2,9-Dimethyl-1,10-phenanthroline)bis[5-methyl-2-(pyridin-2-yl)phenyl]iridium(III) hexafluorophosphate–diethyl ether–acetonitrile (1/0.61/0.78) (2) Data collection

**Table:**

XtaLAB Synergy, Dualflex, HyPix diffractometer	8074 independent reflections
Radiation source: micro-focus sealed X-ray tube, PhotonJet (Cu) X-ray Source	7770 reflections with *I* > 2σ(*I*)
Mirror monochromator	*R*_int_ = 0.058
Detector resolution: 10.0000 pixels mm^-1^	θ_max_ = 80.3°, θ_min_ = 2.8°
ω scans	*h* = −11→10
Absorption correction: multi-scan (CrysAlisPro; Rigaku OD, 2023)	*k* = −16→16
*T*_min_ = 0.509, *T*_max_ = 1.000	*l* = −21→21
62414 measured reflections	

### (2,9-Dimethyl-1,10-phenanthroline)bis[5-methyl-2-(pyridin-2-yl)phenyl]iridium(III) hexafluorophosphate–diethyl ether–acetonitrile (1/0.61/0.78) (2) Refinement

**Table:**

Refinement on *F*^2^	Secondary atom site location: difference Fourier map
Least-squares matrix: full	Hydrogen site location: inferred from neighbouring sites
*R*[*F*^2^ > 2σ(*F*^2^)] = 0.026	H-atom parameters constrained
*wR*(*F*^2^) = 0.067	*w* = 1/[σ^2^(*F*_o_^2^) + (0.0375*P*)^2^ + 2.883*P*] where *P* = (*F*_o_^2^ + 2*F*_c_^2^)/3
*S* = 1.04	(Δ/σ)_max_ = 0.002
8074 reflections	Δρ_max_ = 1.25 e Å^−^^3^
624 parameters	Δρ_min_ = −1.04 e Å^−^^3^
441 restraints	Extinction correction: *SHELXT2018/2* (Sheldrick, 2015a), Fc^*^=kFc[1+0.001xFc^2^λ^3^/sin(2θ)]^-1/4^
Primary atom site location: dual	Extinction coefficient: 0.00030 (4)

### (2,9-Dimethyl-1,10-phenanthroline)bis[5-methyl-2-(pyridin-2-yl)phenyl]iridium(III) hexafluorophosphate–diethyl ether–acetonitrile (1/0.61/0.78) (2) Special details

**Table:**

Geometry. All esds (except the esd in the dihedral angle between two l.s. planes) are estimated using the full covariance matrix. The cell esds are taken into account individually in the estimation of esds in distances, angles and torsion angles; correlations between esds in cell parameters are only used when they are defined by crystal symmetry. An approximate (isotropic) treatment of cell esds is used for estimating esds involving l.s. planes.
Refinement. The PF6 anion is modeled as disordered over two positions (0.645 (6):0.355 (6)). The solvent volume is modeled as a disordered mixture of one diethyl ether and two acetonitrile molecules (0.610 (7):0.390 (7)).Analogous bond lengths and angles among the disordered species were restrained to be similar. Bond lengths for the acetonitriles molecules were restrained toward ideal values. Anisotropic displacement parameters for proximal atoms were restrained to be similar.

### (2,9-Dimethyl-1,10-phenanthroline)bis[5-methyl-2-(pyridin-2-yl)phenyl]iridium(III) hexafluorophosphate–diethyl ether–acetonitrile (1/0.61/0.78) (2) Fractional atomic coordinates and isotropic or equivalent isotropic displacement parameters (Å^2^)

**Table:**

	*x*	*y*	*z*	*U*_iso_*/*U*_eq_	Occ. (<1)
Ir1	0.56177 (2)	0.31354 (2)	0.30009 (2)	0.01467 (5)	
N1	0.7427 (3)	0.40794 (19)	0.29079 (15)	0.0170 (5)	
N2	0.3871 (3)	0.22532 (18)	0.29027 (15)	0.0172 (4)	
N3	0.4473 (3)	0.36887 (19)	0.41150 (15)	0.0166 (4)	
N4	0.6041 (3)	0.18659 (19)	0.41092 (15)	0.0171 (4)	
C1	0.5299 (3)	0.4350 (2)	0.20191 (18)	0.0189 (5)	
C2	0.4143 (3)	0.4469 (2)	0.15642 (18)	0.0203 (6)	
H2	0.339988	0.392279	0.171112	0.024*	
C3	0.4044 (3)	0.5361 (2)	0.09024 (19)	0.0223 (6)	
C4	0.5159 (4)	0.6153 (2)	0.0673 (2)	0.0247 (6)	
H4	0.510492	0.676558	0.022253	0.030*	
C5	0.6338 (4)	0.6052 (2)	0.1094 (2)	0.0238 (6)	
H5	0.709508	0.659043	0.093005	0.029*	
C6	0.6418 (3)	0.5157 (2)	0.17634 (18)	0.0197 (5)	
C7	0.7605 (3)	0.4989 (2)	0.22573 (18)	0.0181 (5)	
C8	0.8847 (3)	0.5650 (2)	0.2112 (2)	0.0234 (6)	
H8	0.897416	0.627800	0.165866	0.028*	
C9	0.9894 (3)	0.5398 (3)	0.2625 (2)	0.0253 (6)	
H9	1.074258	0.584926	0.252567	0.030*	
C10	0.9700 (3)	0.4479 (3)	0.3287 (2)	0.0247 (6)	
H10	1.040461	0.429200	0.364984	0.030*	
C11	0.8450 (3)	0.3842 (2)	0.34052 (19)	0.0208 (6)	
H11	0.831069	0.321103	0.385616	0.025*	
C12	0.2739 (4)	0.5484 (3)	0.0455 (2)	0.0292 (7)	
H12A	0.216237	0.481534	0.062674	0.044*	
H12B	0.309996	0.567350	−0.016366	0.044*	
H12C	0.210703	0.604241	0.060979	0.044*	
C13	0.6590 (3)	0.2572 (2)	0.20116 (18)	0.0192 (5)	
C14	0.8055 (3)	0.2745 (2)	0.15654 (19)	0.0228 (6)	
H14	0.870260	0.318147	0.172469	0.027*	
C15	0.8598 (4)	0.2297 (3)	0.0892 (2)	0.0259 (6)	
C16	0.7631 (4)	0.1681 (3)	0.0646 (2)	0.0291 (7)	
H16	0.798347	0.137079	0.018951	0.035*	
C17	0.6154 (4)	0.1515 (2)	0.1063 (2)	0.0258 (6)	
H17	0.550003	0.110025	0.088789	0.031*	
C18	0.5636 (3)	0.1960 (2)	0.17393 (18)	0.0205 (6)	
C19	0.4123 (3)	0.1793 (2)	0.22387 (18)	0.0194 (5)	
C20	0.2987 (4)	0.1216 (2)	0.2094 (2)	0.0248 (6)	
H20	0.316023	0.089729	0.163159	0.030*	
C21	0.1606 (4)	0.1108 (3)	0.2624 (2)	0.0282 (7)	
H21	0.082658	0.071690	0.252696	0.034*	
C22	0.1369 (3)	0.1574 (3)	0.3299 (2)	0.0260 (6)	
H22	0.042962	0.150330	0.367156	0.031*	
C23	0.2521 (3)	0.2142 (2)	0.34171 (19)	0.0204 (6)	
H23	0.235959	0.246633	0.387628	0.024*	
C24	1.0189 (4)	0.2508 (3)	0.0432 (2)	0.0366 (8)	
H24A	1.031319	0.324148	0.008388	0.055*	
H24B	1.044498	0.202773	0.006432	0.055*	
H24C	1.084347	0.239071	0.084967	0.055*	
C25	0.3716 (3)	0.4571 (2)	0.41300 (19)	0.0188 (5)	
C26	0.3083 (3)	0.4816 (2)	0.4900 (2)	0.0220 (6)	
H26	0.254593	0.544557	0.488837	0.026*	
C27	0.3240 (3)	0.4152 (2)	0.5661 (2)	0.0238 (6)	
H27	0.281885	0.431971	0.617965	0.029*	
C28	0.4026 (3)	0.3221 (2)	0.56737 (19)	0.0213 (6)	
C29	0.4199 (4)	0.2492 (3)	0.64506 (19)	0.0257 (6)	
H29	0.378477	0.263902	0.697809	0.031*	
C30	0.4945 (4)	0.1593 (3)	0.64478 (19)	0.0252 (6)	
H30	0.505359	0.111531	0.697244	0.030*	
C31	0.5569 (3)	0.1356 (2)	0.56666 (19)	0.0211 (6)	
C32	0.6374 (3)	0.0434 (2)	0.5648 (2)	0.0235 (6)	
H32	0.649516	−0.005589	0.616406	0.028*	
C33	0.6974 (3)	0.0250 (2)	0.4888 (2)	0.0214 (6)	
H33	0.752492	−0.036908	0.487234	0.026*	
C34	0.6789 (3)	0.0972 (2)	0.41185 (19)	0.0184 (5)	
C35	0.5433 (3)	0.2058 (2)	0.48826 (18)	0.0176 (5)	
C36	0.4628 (3)	0.3015 (2)	0.48812 (17)	0.0168 (5)	
C37	0.3491 (4)	0.5336 (2)	0.3321 (2)	0.0254 (6)	
H37A	0.445488	0.563933	0.298570	0.038*	
H37B	0.285437	0.590249	0.345523	0.038*	
H37C	0.301724	0.496575	0.299036	0.038*	
C38	0.7485 (4)	0.0714 (2)	0.3307 (2)	0.0256 (6)	
H38A	0.671213	0.064410	0.298840	0.038*	
H38B	0.800504	0.004744	0.343638	0.038*	
H38C	0.819484	0.128252	0.296338	0.038*	
P1'	0.9795 (7)	0.2268 (5)	0.5818 (4)	0.0217 (17)	0.355 (6)
F1'	0.9384 (13)	0.1109 (6)	0.6391 (7)	0.073 (3)	0.355 (6)
F2'	1.0224 (12)	0.3426 (7)	0.5227 (7)	0.075 (3)	0.355 (6)
F3'	0.8153 (6)	0.2633 (7)	0.6055 (8)	0.071 (3)	0.355 (6)
F4'	1.0268 (9)	0.2623 (7)	0.6577 (4)	0.050 (2)	0.355 (6)
F5'	1.1447 (7)	0.1957 (8)	0.5518 (7)	0.065 (3)	0.355 (6)
F6'	0.9316 (9)	0.1941 (8)	0.5040 (4)	0.051 (2)	0.355 (6)
C39	1.0653 (17)	0.0694 (15)	0.8577 (14)	0.083 (4)	0.610 (7)
H39A	1.065509	0.021290	0.821140	0.125*	0.610 (7)
H39B	1.044449	0.028852	0.917608	0.125*	0.610 (7)
H39C	1.162689	0.105539	0.844568	0.125*	0.610 (7)
C40	0.9449 (9)	0.1517 (6)	0.8415 (5)	0.0510 (19)	0.610 (7)
H40A	0.952585	0.204385	0.873883	0.061*	0.610 (7)
H40B	0.963505	0.189339	0.780110	0.061*	0.610 (7)
O1	0.7975 (7)	0.1062 (4)	0.8654 (3)	0.0537 (16)	0.610 (7)
C41	0.6940 (18)	0.1884 (13)	0.8387 (14)	0.072 (3)	0.610 (7)
H41A	0.711661	0.214101	0.775799	0.087*	0.610 (7)
H41B	0.704928	0.248940	0.862746	0.087*	0.610 (7)
C42	0.5369 (11)	0.1352 (9)	0.8739 (7)	0.056 (2)	0.610 (7)
H42A	0.530344	0.072823	0.852377	0.083*	0.610 (7)
H42B	0.461848	0.185540	0.855091	0.083*	0.610 (7)
H42C	0.519384	0.113749	0.936317	0.083*	0.610 (7)
N5'	0.8946 (17)	0.0117 (13)	0.9021 (11)	0.096 (5)	0.390 (7)
N6'	0.693 (3)	0.205 (2)	0.831 (3)	0.105 (6)	0.390 (7)
C39'	1.0100 (19)	0.0516 (19)	0.8725 (15)	0.055 (4)	0.390 (7)
C40'	1.1573 (18)	0.1031 (13)	0.8375 (15)	0.103 (7)	0.390 (7)
H40C	1.150402	0.164271	0.789652	0.154*	0.390 (7)
H40D	1.226436	0.052786	0.817689	0.154*	0.390 (7)
H40E	1.193331	0.126654	0.881952	0.154*	0.390 (7)
C41'	0.600 (3)	0.144 (2)	0.8690 (18)	0.086 (5)	0.390 (7)
C42'	0.447 (3)	0.1071 (19)	0.9099 (18)	0.129 (7)	0.390 (7)
H42D	0.379334	0.136728	0.870853	0.193*	0.390 (7)
H42E	0.417843	0.129945	0.962383	0.193*	0.390 (7)
H42F	0.440850	0.029678	0.923506	0.193*	0.390 (7)
P1	0.9720 (4)	0.2211 (3)	0.5773 (2)	0.0221 (9)	0.645 (6)
F1	0.8599 (5)	0.1457 (3)	0.6530 (2)	0.0385 (10)	0.645 (6)
F2	1.0832 (4)	0.2965 (4)	0.4991 (2)	0.0412 (10)	0.645 (6)
F3	0.8362 (4)	0.2690 (3)	0.5333 (3)	0.0475 (12)	0.645 (6)
F4	0.9609 (5)	0.3118 (3)	0.6251 (3)	0.0410 (10)	0.645 (6)
F5	1.1101 (5)	0.1733 (3)	0.6173 (4)	0.0562 (15)	0.645 (6)
F6	0.9824 (5)	0.1322 (4)	0.5268 (3)	0.0537 (13)	0.645 (6)

### (2,9-Dimethyl-1,10-phenanthroline)bis[5-methyl-2-(pyridin-2-yl)phenyl]iridium(III) hexafluorophosphate–diethyl ether–acetonitrile (1/0.61/0.78) (2) Atomic displacement parameters (Å^2^)

**Table:**

	*U*^11^	*U*^22^	*U*^33^	*U*^12^	*U*^13^	*U*^23^
Ir1	0.01239 (7)	0.01474 (7)	0.01579 (7)	0.00190 (4)	−0.00104 (4)	−0.00338 (4)
N1	0.0139 (11)	0.0181 (11)	0.0175 (11)	0.0000 (9)	0.0007 (9)	−0.0044 (9)
N2	0.0164 (11)	0.0158 (11)	0.0191 (11)	0.0020 (9)	−0.0037 (9)	−0.0039 (9)
N3	0.0124 (10)	0.0192 (11)	0.0183 (11)	0.0007 (9)	−0.0003 (9)	−0.0072 (9)
N4	0.0129 (11)	0.0177 (11)	0.0192 (11)	−0.0006 (9)	−0.0026 (9)	−0.0028 (9)
C1	0.0192 (13)	0.0199 (13)	0.0166 (12)	0.0037 (11)	−0.0003 (10)	−0.0056 (10)
C2	0.0196 (14)	0.0213 (14)	0.0194 (13)	0.0020 (11)	−0.0017 (11)	−0.0060 (11)
C3	0.0240 (15)	0.0257 (15)	0.0181 (13)	0.0087 (12)	−0.0056 (11)	−0.0067 (11)
C4	0.0269 (16)	0.0230 (14)	0.0211 (14)	0.0058 (12)	−0.0030 (12)	−0.0017 (11)
C5	0.0246 (15)	0.0206 (14)	0.0235 (14)	0.0007 (11)	−0.0006 (12)	−0.0039 (11)
C6	0.0187 (14)	0.0216 (14)	0.0182 (13)	0.0032 (11)	−0.0010 (11)	−0.0061 (11)
C7	0.0171 (13)	0.0196 (13)	0.0164 (12)	0.0031 (10)	0.0010 (10)	−0.0057 (10)
C8	0.0210 (14)	0.0219 (14)	0.0244 (14)	−0.0021 (11)	−0.0002 (11)	−0.0041 (11)
C9	0.0170 (14)	0.0272 (15)	0.0302 (16)	−0.0042 (11)	−0.0008 (12)	−0.0073 (13)
C10	0.0169 (14)	0.0284 (15)	0.0288 (15)	0.0017 (11)	−0.0063 (12)	−0.0064 (12)
C11	0.0168 (13)	0.0235 (14)	0.0207 (13)	0.0012 (11)	−0.0019 (11)	−0.0047 (11)
C12	0.0315 (17)	0.0307 (16)	0.0257 (15)	0.0075 (13)	−0.0094 (13)	−0.0056 (13)
C13	0.0216 (14)	0.0175 (13)	0.0167 (12)	0.0056 (11)	−0.0013 (11)	−0.0035 (10)
C14	0.0233 (15)	0.0206 (14)	0.0211 (14)	0.0036 (11)	−0.0018 (11)	−0.0016 (11)
C15	0.0232 (15)	0.0266 (15)	0.0215 (14)	0.0074 (12)	0.0049 (12)	−0.0025 (12)
C16	0.0341 (18)	0.0252 (15)	0.0258 (15)	0.0069 (13)	0.0028 (13)	−0.0095 (13)
C17	0.0306 (17)	0.0233 (14)	0.0230 (14)	0.0040 (12)	−0.0035 (12)	−0.0065 (12)
C18	0.0215 (14)	0.0202 (13)	0.0188 (13)	0.0036 (11)	−0.0027 (11)	−0.0043 (11)
C19	0.0209 (14)	0.0182 (13)	0.0184 (13)	0.0038 (11)	−0.0040 (11)	−0.0035 (10)
C20	0.0246 (15)	0.0245 (15)	0.0295 (15)	0.0046 (12)	−0.0089 (12)	−0.0118 (12)
C21	0.0203 (15)	0.0326 (17)	0.0363 (17)	−0.0006 (12)	−0.0095 (13)	−0.0138 (14)
C22	0.0162 (14)	0.0314 (16)	0.0306 (16)	0.0009 (12)	−0.0035 (12)	−0.0094 (13)
C23	0.0185 (14)	0.0211 (13)	0.0201 (13)	0.0024 (11)	−0.0026 (11)	−0.0038 (11)
C24	0.0290 (18)	0.045 (2)	0.0305 (17)	0.0073 (15)	0.0062 (14)	−0.0103 (15)
C25	0.0126 (12)	0.0208 (13)	0.0232 (14)	0.0013 (10)	−0.0026 (10)	−0.0066 (11)
C26	0.0155 (13)	0.0254 (14)	0.0273 (15)	0.0020 (11)	−0.0002 (11)	−0.0140 (12)
C27	0.0203 (14)	0.0283 (15)	0.0237 (14)	−0.0007 (12)	0.0013 (11)	−0.0121 (12)
C28	0.0178 (14)	0.0255 (15)	0.0212 (14)	−0.0012 (11)	−0.0012 (11)	−0.0085 (12)
C29	0.0283 (16)	0.0321 (16)	0.0160 (13)	−0.0029 (13)	0.0007 (12)	−0.0084 (12)
C30	0.0281 (16)	0.0278 (15)	0.0171 (13)	−0.0032 (12)	−0.0045 (12)	−0.0006 (11)
C31	0.0168 (13)	0.0226 (14)	0.0235 (14)	−0.0020 (11)	−0.0054 (11)	−0.0036 (11)
C32	0.0230 (15)	0.0209 (14)	0.0253 (15)	−0.0007 (11)	−0.0095 (12)	−0.0003 (11)
C33	0.0165 (13)	0.0168 (13)	0.0298 (15)	0.0014 (10)	−0.0076 (11)	−0.0021 (11)
C34	0.0131 (12)	0.0160 (12)	0.0245 (14)	0.0004 (10)	−0.0036 (10)	−0.0025 (11)
C35	0.0164 (13)	0.0188 (13)	0.0173 (13)	−0.0034 (10)	−0.0024 (10)	−0.0045 (10)
C36	0.0147 (12)	0.0175 (13)	0.0163 (12)	−0.0024 (10)	−0.0014 (10)	−0.0020 (10)
C37	0.0274 (16)	0.0254 (15)	0.0251 (15)	0.0117 (12)	−0.0066 (12)	−0.0095 (12)
C38	0.0254 (15)	0.0228 (14)	0.0254 (15)	0.0097 (12)	−0.0021 (12)	−0.0035 (12)
P1'	0.017 (3)	0.019 (3)	0.031 (3)	0.0037 (18)	−0.007 (2)	−0.008 (2)
F1'	0.073 (6)	0.034 (4)	0.101 (7)	−0.025 (4)	−0.053 (6)	0.030 (4)
F2'	0.076 (7)	0.051 (5)	0.087 (7)	−0.033 (5)	−0.045 (6)	0.024 (5)
F3'	0.019 (3)	0.077 (5)	0.140 (9)	0.010 (3)	−0.006 (4)	−0.076 (6)
F4'	0.044 (4)	0.075 (6)	0.040 (4)	−0.021 (4)	−0.004 (3)	−0.031 (4)
F5'	0.023 (3)	0.095 (6)	0.099 (7)	0.006 (4)	−0.004 (4)	−0.071 (6)
F6'	0.040 (4)	0.080 (6)	0.033 (3)	−0.020 (4)	−0.009 (3)	−0.014 (4)
C39	0.076 (11)	0.071 (9)	0.092 (11)	0.025 (8)	−0.012 (9)	−0.007 (8)
C40	0.066 (5)	0.050 (4)	0.036 (3)	−0.004 (4)	−0.009 (3)	−0.009 (3)
O1	0.087 (4)	0.034 (3)	0.044 (3)	0.007 (2)	−0.018 (3)	−0.013 (2)
C41	0.072 (7)	0.085 (7)	0.075 (7)	0.012 (6)	−0.012 (6)	−0.052 (6)
C42	0.054 (6)	0.068 (5)	0.052 (5)	0.005 (5)	−0.005 (5)	−0.031 (4)
N5'	0.112 (12)	0.094 (10)	0.091 (10)	0.022 (9)	−0.031 (9)	−0.033 (8)
N6'	0.094 (10)	0.119 (12)	0.100 (11)	0.029 (10)	0.003 (9)	−0.043 (10)
C39'	0.058 (11)	0.057 (9)	0.049 (8)	0.018 (8)	0.001 (8)	−0.022 (7)
C40'	0.078 (11)	0.054 (9)	0.124 (13)	0.012 (8)	0.052 (10)	0.017 (9)
C41'	0.086 (9)	0.102 (9)	0.078 (8)	0.001 (9)	−0.005 (9)	−0.047 (7)
C42'	0.144 (16)	0.099 (13)	0.142 (16)	−0.027 (13)	0.007 (14)	−0.052 (12)
P1	0.0177 (15)	0.0212 (15)	0.0277 (15)	0.0012 (10)	−0.0015 (11)	−0.0088 (11)
F1	0.043 (2)	0.039 (2)	0.0278 (16)	−0.0170 (17)	−0.0064 (16)	0.0027 (15)
F2	0.030 (2)	0.056 (3)	0.0344 (18)	−0.0180 (18)	0.0032 (15)	−0.0114 (17)
F3	0.0315 (19)	0.044 (2)	0.059 (3)	−0.0056 (15)	−0.0176 (18)	0.0081 (19)
F4	0.041 (2)	0.0363 (19)	0.050 (2)	−0.0052 (16)	0.0051 (17)	−0.0285 (18)
F5	0.036 (2)	0.0312 (18)	0.103 (4)	0.0065 (15)	−0.041 (3)	−0.003 (2)
F6	0.042 (2)	0.057 (3)	0.073 (3)	−0.010 (2)	0.003 (2)	−0.046 (3)

### (2,9-Dimethyl-1,10-phenanthroline)bis[5-methyl-2-(pyridin-2-yl)phenyl]iridium(III) hexafluorophosphate–diethyl ether–acetonitrile (1/0.61/0.78) (2) Geometric parameters (Å, º)

**Table:**

Ir1—N1	2.050 (2)	C26—H26	0.9500
Ir1—N2	2.051 (2)	C26—C27	1.362 (5)
Ir1—N3	2.226 (2)	C27—H27	0.9500
Ir1—N4	2.222 (2)	C27—C28	1.401 (4)
Ir1—C1	2.018 (3)	C28—C29	1.422 (4)
Ir1—C13	2.017 (3)	C28—C36	1.414 (4)
N1—C7	1.371 (4)	C29—H29	0.9500
N1—C11	1.343 (4)	C29—C30	1.349 (5)
N2—C19	1.366 (4)	C30—H30	0.9500
N2—C23	1.349 (4)	C30—C31	1.420 (4)
N3—C25	1.339 (4)	C31—C32	1.406 (4)
N3—C36	1.374 (4)	C31—C35	1.407 (4)
N4—C34	1.343 (4)	C32—H32	0.9500
N4—C35	1.380 (4)	C32—C33	1.353 (5)
C1—C2	1.397 (4)	C33—H33	0.9500
C1—C6	1.414 (4)	C33—C34	1.412 (4)
C2—H2	0.9500	C34—C38	1.492 (4)
C2—C3	1.393 (4)	C35—C36	1.441 (4)
C3—C4	1.400 (5)	C37—H37A	0.9800
C3—C12	1.509 (4)	C37—H37B	0.9800
C4—H4	0.9500	C37—H37C	0.9800
C4—C5	1.382 (5)	C38—H38A	0.9800
C5—H5	0.9500	C38—H38B	0.9800
C5—C6	1.399 (4)	C38—H38C	0.9800
C6—C7	1.462 (4)	P1'—F1'	1.576 (6)
C7—C8	1.392 (4)	P1'—F2'	1.587 (6)
C8—H8	0.9500	P1'—F3'	1.579 (6)
C8—C9	1.380 (5)	P1'—F4'	1.583 (6)
C9—H9	0.9500	P1'—F5'	1.581 (6)
C9—C10	1.389 (4)	P1'—F6'	1.595 (6)
C10—H10	0.9500	C39—H39A	0.9800
C10—C11	1.386 (4)	C39—H39B	0.9800
C11—H11	0.9500	C39—H39C	0.9800
C12—H12A	0.9800	C39—C40	1.538 (17)
C12—H12B	0.9800	C40—H40A	0.9900
C12—H12C	0.9800	C40—H40B	0.9900
C13—C14	1.396 (4)	C40—O1	1.431 (10)
C13—C18	1.408 (4)	O1—C41	1.457 (18)
C14—H14	0.9500	C41—H41A	0.9900
C14—C15	1.396 (4)	C41—H41B	0.9900
C15—C16	1.395 (5)	C41—C42	1.553 (17)
C15—C24	1.507 (5)	C42—H42A	0.9800
C16—H16	0.9500	C42—H42B	0.9800
C16—C17	1.392 (5)	C42—H42C	0.9800
C17—H17	0.9500	N5'—C39'	1.152 (9)
C17—C18	1.394 (4)	N6'—C41'	1.154 (10)
C18—C19	1.462 (4)	C39'—C40'	1.468 (9)
C19—C20	1.393 (4)	C40'—H40C	0.9800
C20—H20	0.9500	C40'—H40D	0.9800
C20—C21	1.383 (5)	C40'—H40E	0.9800
C21—H21	0.9500	C41'—C42'	1.471 (10)
C21—C22	1.387 (5)	C42'—H42D	0.9800
C22—H22	0.9500	C42'—H42E	0.9800
C22—C23	1.378 (4)	C42'—H42F	0.9800
C23—H23	0.9500	P1—F1	1.585 (4)
C24—H24A	0.9800	P1—F2	1.600 (4)
C24—H24B	0.9800	P1—F3	1.597 (4)
C24—H24C	0.9800	P1—F4	1.582 (4)
C25—C26	1.409 (4)	P1—F5	1.583 (4)
C25—C37	1.496 (4)	P1—F6	1.593 (4)
			
N1—Ir1—N2	171.61 (9)	C26—C27—H27	120.2
N1—Ir1—N3	89.41 (9)	C26—C27—C28	119.6 (3)
N1—Ir1—N4	96.50 (9)	C28—C27—H27	120.2
N2—Ir1—N3	97.19 (9)	C27—C28—C29	121.8 (3)
N2—Ir1—N4	90.07 (9)	C27—C28—C36	117.6 (3)
N4—Ir1—N3	76.79 (9)	C36—C28—C29	120.6 (3)
C1—Ir1—N1	80.42 (11)	C28—C29—H29	119.6
C1—Ir1—N2	93.13 (11)	C30—C29—C28	120.8 (3)
C1—Ir1—N3	101.51 (10)	C30—C29—H29	119.6
C1—Ir1—N4	176.54 (10)	C29—C30—H30	119.8
C13—Ir1—N1	93.12 (11)	C29—C30—C31	120.5 (3)
C13—Ir1—N2	80.39 (11)	C31—C30—H30	119.8
C13—Ir1—N3	177.26 (10)	C32—C31—C30	121.5 (3)
C13—Ir1—N4	101.85 (10)	C32—C31—C35	117.9 (3)
C13—Ir1—C1	79.97 (11)	C35—C31—C30	120.6 (3)
C7—N1—Ir1	115.65 (19)	C31—C32—H32	120.3
C11—N1—Ir1	124.8 (2)	C33—C32—C31	119.5 (3)
C11—N1—C7	119.5 (2)	C33—C32—H32	120.3
C19—N2—Ir1	115.46 (19)	C32—C33—H33	119.7
C23—N2—Ir1	124.8 (2)	C32—C33—C34	120.5 (3)
C23—N2—C19	119.7 (2)	C34—C33—H33	119.7
C25—N3—Ir1	129.3 (2)	N4—C34—C33	121.8 (3)
C25—N3—C36	117.9 (2)	N4—C34—C38	120.8 (3)
C36—N3—Ir1	112.78 (18)	C33—C34—C38	117.3 (3)
C34—N4—Ir1	129.1 (2)	N4—C35—C31	122.5 (3)
C34—N4—C35	117.8 (2)	N4—C35—C36	118.4 (2)
C35—N4—Ir1	113.05 (18)	C31—C35—C36	119.2 (3)
C2—C1—Ir1	128.0 (2)	N3—C36—C28	122.7 (3)
C2—C1—C6	117.5 (3)	N3—C36—C35	119.0 (2)
C6—C1—Ir1	114.4 (2)	C28—C36—C35	118.3 (3)
C1—C2—H2	118.9	C25—C37—H37A	109.5
C3—C2—C1	122.2 (3)	C25—C37—H37B	109.5
C3—C2—H2	118.9	C25—C37—H37C	109.5
C2—C3—C4	118.8 (3)	H37A—C37—H37B	109.5
C2—C3—C12	120.6 (3)	H37A—C37—H37C	109.5
C4—C3—C12	120.6 (3)	H37B—C37—H37C	109.5
C3—C4—H4	119.7	C34—C38—H38A	109.5
C5—C4—C3	120.6 (3)	C34—C38—H38B	109.5
C5—C4—H4	119.7	C34—C38—H38C	109.5
C4—C5—H5	120.0	H38A—C38—H38B	109.5
C4—C5—C6	120.0 (3)	H38A—C38—H38C	109.5
C6—C5—H5	120.0	H38B—C38—H38C	109.5
C1—C6—C7	115.2 (3)	F1'—P1'—F2'	179.0 (8)
C5—C6—C1	120.8 (3)	F1'—P1'—F3'	91.8 (6)
C5—C6—C7	124.0 (3)	F1'—P1'—F4'	92.1 (6)
N1—C7—C6	114.2 (2)	F1'—P1'—F5'	91.4 (6)
N1—C7—C8	119.9 (3)	F1'—P1'—F6'	89.3 (6)
C8—C7—C6	126.0 (3)	F2'—P1'—F6'	89.9 (6)
C7—C8—H8	119.9	F3'—P1'—F2'	88.9 (6)
C9—C8—C7	120.3 (3)	F3'—P1'—F4'	91.7 (5)
C9—C8—H8	119.9	F3'—P1'—F5'	176.1 (7)
C8—C9—H9	120.2	F3'—P1'—F6'	87.9 (5)
C8—C9—C10	119.5 (3)	F4'—P1'—F2'	88.7 (6)
C10—C9—H9	120.2	F4'—P1'—F6'	178.6 (6)
C9—C10—H10	120.9	F5'—P1'—F2'	87.8 (6)
C11—C10—C9	118.2 (3)	F5'—P1'—F4'	90.4 (5)
C11—C10—H10	120.9	F5'—P1'—F6'	90.0 (5)
N1—C11—C10	122.7 (3)	H39A—C39—H39B	109.5
N1—C11—H11	118.7	H39A—C39—H39C	109.5
C10—C11—H11	118.7	H39B—C39—H39C	109.5
C3—C12—H12A	109.5	C40—C39—H39A	109.5
C3—C12—H12B	109.5	C40—C39—H39B	109.5
C3—C12—H12C	109.5	C40—C39—H39C	109.5
H12A—C12—H12B	109.5	C39—C40—H40A	108.9
H12A—C12—H12C	109.5	C39—C40—H40B	108.9
H12B—C12—H12C	109.5	H40A—C40—H40B	107.7
C14—C13—Ir1	127.8 (2)	O1—C40—C39	113.2 (9)
C14—C13—C18	117.8 (3)	O1—C40—H40A	108.9
C18—C13—Ir1	114.4 (2)	O1—C40—H40B	108.9
C13—C14—H14	119.0	C40—O1—C41	108.8 (7)
C13—C14—C15	122.1 (3)	O1—C41—H41A	110.7
C15—C14—H14	119.0	O1—C41—H41B	110.7
C14—C15—C24	120.0 (3)	O1—C41—C42	105.4 (11)
C16—C15—C14	118.7 (3)	H41A—C41—H41B	108.8
C16—C15—C24	121.3 (3)	C42—C41—H41A	110.7
C15—C16—H16	119.7	C42—C41—H41B	110.7
C17—C16—C15	120.6 (3)	C41—C42—H42A	109.5
C17—C16—H16	119.7	C41—C42—H42B	109.5
C16—C17—H17	120.1	C41—C42—H42C	109.5
C16—C17—C18	119.8 (3)	H42A—C42—H42B	109.5
C18—C17—H17	120.1	H42A—C42—H42C	109.5
C13—C18—C19	115.3 (3)	H42B—C42—H42C	109.5
C17—C18—C13	120.9 (3)	N5'—C39'—C40'	178 (2)
C17—C18—C19	123.7 (3)	C39'—C40'—H40C	109.5
N2—C19—C18	114.3 (3)	C39'—C40'—H40D	109.5
N2—C19—C20	119.9 (3)	C39'—C40'—H40E	109.5
C20—C19—C18	125.8 (3)	H40C—C40'—H40D	109.5
C19—C20—H20	120.1	H40C—C40'—H40E	109.5
C21—C20—C19	119.9 (3)	H40D—C40'—H40E	109.5
C21—C20—H20	120.1	N6'—C41'—C42'	156 (3)
C20—C21—H21	120.2	C41'—C42'—H42D	109.5
C20—C21—C22	119.5 (3)	C41'—C42'—H42E	109.5
C22—C21—H21	120.2	C41'—C42'—H42F	109.5
C21—C22—H22	120.6	H42D—C42'—H42E	109.5
C23—C22—C21	118.7 (3)	H42D—C42'—H42F	109.5
C23—C22—H22	120.6	H42E—C42'—H42F	109.5
N2—C23—C22	122.2 (3)	F1—P1—F2	178.5 (3)
N2—C23—H23	118.9	F1—P1—F3	90.0 (3)
C22—C23—H23	118.9	F1—P1—F6	88.8 (3)
C15—C24—H24A	109.5	F3—P1—F2	89.0 (3)
C15—C24—H24B	109.5	F4—P1—F1	92.2 (3)
C15—C24—H24C	109.5	F4—P1—F2	88.9 (3)
H24A—C24—H24B	109.5	F4—P1—F3	90.2 (3)
H24A—C24—H24C	109.5	F4—P1—F5	91.4 (3)
H24B—C24—H24C	109.5	F4—P1—F6	178.4 (4)
N3—C25—C26	122.0 (3)	F5—P1—F1	91.6 (3)
N3—C25—C37	120.8 (3)	F5—P1—F2	89.4 (3)
C26—C25—C37	117.2 (3)	F5—P1—F3	177.7 (4)
C25—C26—H26	119.9	F5—P1—F6	89.8 (3)
C27—C26—C25	120.3 (3)	F6—P1—F2	90.1 (3)
C27—C26—H26	119.9	F6—P1—F3	88.6 (3)
			
Ir1—N1—C7—C6	3.0 (3)	C14—C13—C18—C19	179.1 (3)
Ir1—N1—C7—C8	−176.3 (2)	C14—C15—C16—C17	0.0 (5)
Ir1—N1—C11—C10	176.3 (2)	C15—C16—C17—C18	−0.6 (5)
Ir1—N2—C19—C18	3.4 (3)	C16—C17—C18—C13	−0.4 (5)
Ir1—N2—C19—C20	−177.2 (2)	C16—C17—C18—C19	−177.3 (3)
Ir1—N2—C23—C22	177.2 (2)	C17—C18—C19—N2	176.5 (3)
Ir1—N3—C25—C26	−178.4 (2)	C17—C18—C19—C20	−2.8 (5)
Ir1—N3—C25—C37	2.4 (4)	C18—C13—C14—C15	−2.7 (4)
Ir1—N3—C36—C28	178.3 (2)	C18—C19—C20—C21	179.1 (3)
Ir1—N3—C36—C35	−1.8 (3)	C19—N2—C23—C22	0.1 (4)
Ir1—N4—C34—C33	179.0 (2)	C19—C20—C21—C22	−0.2 (5)
Ir1—N4—C34—C38	0.2 (4)	C20—C21—C22—C23	0.5 (5)
Ir1—N4—C35—C31	−180.0 (2)	C21—C22—C23—N2	−0.4 (5)
Ir1—N4—C35—C36	0.2 (3)	C23—N2—C19—C18	−179.1 (2)
Ir1—C1—C2—C3	179.1 (2)	C23—N2—C19—C20	0.2 (4)
Ir1—C1—C6—C5	−179.6 (2)	C24—C15—C16—C17	−178.4 (3)
Ir1—C1—C6—C7	−0.9 (3)	C25—N3—C36—C28	−0.2 (4)
Ir1—C13—C14—C15	179.4 (2)	C25—N3—C36—C35	179.8 (2)
Ir1—C13—C18—C17	−179.8 (2)	C25—C26—C27—C28	−0.4 (4)
Ir1—C13—C18—C19	−2.7 (3)	C26—C27—C28—C29	−178.8 (3)
N1—C7—C8—C9	−0.5 (4)	C26—C27—C28—C36	0.0 (4)
N2—C19—C20—C21	−0.1 (5)	C27—C28—C29—C30	179.2 (3)
N3—C25—C26—C27	0.5 (4)	C27—C28—C36—N3	0.2 (4)
N4—C35—C36—N3	1.1 (4)	C27—C28—C36—C35	−179.7 (3)
N4—C35—C36—C28	−179.0 (2)	C28—C29—C30—C31	−0.2 (5)
C1—C2—C3—C4	1.6 (4)	C29—C28—C36—N3	179.1 (3)
C1—C2—C3—C12	−177.0 (3)	C29—C28—C36—C35	−0.8 (4)
C1—C6—C7—N1	−1.4 (4)	C29—C30—C31—C32	178.8 (3)
C1—C6—C7—C8	177.9 (3)	C29—C30—C31—C35	0.6 (5)
C2—C1—C6—C5	1.8 (4)	C30—C31—C32—C33	−178.7 (3)
C2—C1—C6—C7	−179.6 (2)	C30—C31—C35—N4	179.0 (3)
C2—C3—C4—C5	0.0 (5)	C30—C31—C35—C36	−1.1 (4)
C3—C4—C5—C6	−0.6 (5)	C31—C32—C33—C34	−0.4 (4)
C4—C5—C6—C1	−0.3 (4)	C31—C35—C36—N3	−178.8 (2)
C4—C5—C6—C7	−178.8 (3)	C31—C35—C36—C28	1.2 (4)
C5—C6—C7—N1	177.2 (3)	C32—C31—C35—N4	0.8 (4)
C5—C6—C7—C8	−3.5 (5)	C32—C31—C35—C36	−179.4 (3)
C6—C1—C2—C3	−2.4 (4)	C32—C33—C34—N4	1.1 (4)
C6—C7—C8—C9	−179.7 (3)	C32—C33—C34—C38	180.0 (3)
C7—N1—C11—C10	−0.4 (4)	C34—N4—C35—C31	−0.2 (4)
C7—C8—C9—C10	0.0 (5)	C34—N4—C35—C36	180.0 (2)
C8—C9—C10—C11	0.3 (5)	C35—N4—C34—C33	−0.8 (4)
C9—C10—C11—N1	−0.1 (5)	C35—N4—C34—C38	−179.6 (3)
C11—N1—C7—C6	180.0 (2)	C35—C31—C32—C33	−0.4 (4)
C11—N1—C7—C8	0.7 (4)	C36—N3—C25—C26	−0.2 (4)
C12—C3—C4—C5	178.6 (3)	C36—N3—C25—C37	−179.4 (3)
C13—C14—C15—C16	1.7 (5)	C36—C28—C29—C30	0.3 (5)
C13—C14—C15—C24	−179.9 (3)	C37—C25—C26—C27	179.8 (3)
C13—C18—C19—N2	−0.5 (4)	C39—C40—O1—C41	−173.7 (13)
C13—C18—C19—C20	−179.8 (3)	C40—O1—C41—C42	−175.2 (10)
C14—C13—C18—C17	2.0 (4)		

### (2,9-Dimethyl-1,10-phenanthroline)bis[5-methyl-2-(pyridin-2-yl)phenyl]iridium(III) hexafluorophosphate–diethyl ether–acetonitrile (1/0.61/0.78) (2) Hydrogen-bond geometry (Å, º)

**Table:**

*D*—H···*A*	*D*—H	H···*A*	*D*···*A*	*D*—H···*A*
C10—H10···F2′	0.95	2.54	3.270 (9)	134
C10—H10···F2	0.95	2.53	3.329 (5)	142
C11—H11···F6′	0.95	2.53	3.373 (9)	149
C11—H11···F3	0.95	2.37	3.140 (5)	138
C22—H22···F6′^i^	0.95	2.51	3.272 (8)	138
C23—H23···F2^i^	0.95	2.32	3.194 (5)	152
C26—H26···F3^ii^	0.95	2.52	3.455 (5)	168
C33—H33···F5′^iii^	0.95	2.45	3.372 (8)	163
C37—H37*B*···F3′^ii^	0.98	2.37	3.330 (7)	165
C38—H38*B*···F5^iii^	0.98	2.42	3.393 (5)	171

Symmetry codes: (i) *x*−1, *y*, *z*; (ii) −*x*+1, −*y*+1, −*z*+1; (iii) −*x*+2, −*y*, −*z*+1.
